# Immunological imprint of COVID‐19 on human peripheral blood leukocyte populations

**DOI:** 10.1111/all.14647

**Published:** 2020-11-22

**Authors:** Bernhard Kratzer, Doris Trapin, Paul Ettel, Ulrike Körmöczi, Arno Rottal, Friedrich Tuppy, Melanie Feichter, Pia Gattinger, Kristina Borochova, Yulia Dorofeeva, Inna Tulaeva, Milena Weber, Katharina Grabmeier‐Pfistershammer, Peter A. Tauber, Marika Gerdov, Bernhard Mühl, Thomas Perkmann, Ingrid Fae, Sabine Wenda, Harald Führer, Rainer Henning, Rudolf Valenta, Winfried F. Pickl

**Affiliations:** ^1^ Institute of Immunology Center for Pathophysiology, Infectiology and Immunology Medical University of Vienna Vienna Austria; ^2^ Institute of Pathophysiology and Allergy Research Center for Pathophysiology, Infectiology and Immunology Medical University of Vienna Vienna Austria; ^3^ Laboratory for Immunopathology Department of Clinical Immunology and Allergology I. M. Sechenov First Moscow State Medical University (Sechenov University) Moscow Russia; ^4^ Department of Laboratory Medicine Medical University of Vienna Vienna Austria; ^5^ Labors.at Vienna Austria; ^6^ Department for Blood Group Serology and Transfusion Medicine Medical University of Vienna Vienna Austria; ^7^ Statistical Consultants Vienna Austria; ^8^ Viravaxx Vienna Austria; ^9^ NRC Institute of Immunology FMBA of Russia Moscow Russia; ^10^ Karl Landsteiner University of Health Sciences Krems Austria

**Keywords:** B cells, clinical immunology, coronavirus disease 2019, flow cytometry, infections, lymphocytes, SARS‐CoV‐2, T cells

## Abstract

**Background:**

SARS‐CoV‐2 has triggered a pandemic that is now claiming many lives. Several studies have investigated cellular immune responses in COVID‐19‐infected patients during disease but little is known regarding a possible protracted impact of COVID‐19 on the adaptive and innate immune system in COVID‐19 convalescent patients.

**Methods:**

We used multiparametric flow cytometry to analyze whole peripheral blood samples and determined SARS‐CoV‐2‐specific antibody levels against the S‐protein, its RBD‐subunit, and viral nucleocapsid in a cohort of COVID‐19 convalescent patients who had mild disease ~10 weeks after infection (n = 109) and healthy control subjects (n = 98). Furthermore, we correlated immunological changes with clinical and demographic parameters.

**Results:**

Even ten weeks after disease COVID‐19 convalescent patients had fewer neutrophils, while their cytotoxic CD8^+^ T cells were activated, reflected as higher HLA‐DR and CD38 expression. Multiparametric regression analyses showed that in COVID‐19‐infected patients both CD3^+^CD4^+^ and CD3^+^CD8^+^ effector memory cells were higher, while CD25^+^Foxp3^+^ T regulatory cells were lower. In addition, both transitional B cell and plasmablast levels were significantly elevated in COVID‐19‐infected patients. Fever (duration, level) correlated with numbers of central memory CD4^+^ T cells and anti‐S and anti‐RBD, but not anti‐NC antibody levels. Moreover, a “young immunological age” as determined by numbers of CD3^+^CD45RA^+^CD62L^+^CD31^+^ recent thymic emigrants was associated with a loss of sense of taste and/or smell.

**Conclusion:**

Acute SARS‐CoV‐2 infection leaves protracted beneficial (ie, activation of T cells) and potentially harmful (ie, reduction of neutrophils) imprints in the cellular immune system in addition to induction of specific antibody responses.

## Graphical abstract

Ten weeks after disease, COVID‐19 patients had fewer neutrophils compared to subjects without COVID‐19, while their cytotoxic CD8^+^ T cells were still activated. In COVID‐19 patients both CD3^+^CD4^+^ and CD3^+^CD8^+^ effector memory cells, transitional B cells and plasmablast levels were higher, while CD25^+^Foxp3^+^ T regulatory cells were lower than in subjects without COVID‐19. Fever duration correlated with higher numbers of central memory CD4^+^ T cells, anti‐S and anti‐RBD antibody levels, while loss of taste/smell was associated with higher levels of recent thymic emigrants.

Abbreviations: COVID‐19, coronavirus disease 2019; ELISA, enzyme‐linked immunosorbent assay; KRECs, kappa‐deleting recombination excision circles; qPCR, quantitative PCR; RBD, receptor‐binding domain of SARS‐CoV2 spike protein; TRECs, T‐cell receptor excision circles; S, spike protein of SARS‐Cov2; SARS‐CoV‐2, severe acute respiratory syndrome coronavirus 2.

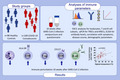

**Abbreviations**
ACE2: angiotensin converting enzyme 2
CD: cluster of differentiation
CoV‐2: coronavirus 2
COVID‐19: coronavirus disease 2019
EBV: Epstein‐Barr virus
ELISA: enzyme‐linked immunosorbent assay
HC: healthy control
HLA: human leukocyte antigen
HRP: horseradish peroxidase
Ig: immunoglobulin
KREC: kappa‐deleting recombination excision circles
MERS: Middle East respiratory syndrome
NC: nucleocapsid
OD: optical density
PB: peripheral blood
PBMC: peripheral blood mononuclear cells
PCA: principal component analysis
RBD: receptor‐binding domain
RTE: recent thymic emigrant T cells
rtPCR: reverse transcription polymerase chain reaction
S: spike protein
SARS: severe acute respiratory syndrome
TRAC: T cell receptor alpha chain
TREC: T cell receptor excision circles

## 1 INTRODUCTION

An outbreak of an epidemic respiratory infection that caused severe pneumonia in a subgroup of patients was reported in December 2019 in Wuhan City, Hubei Province, China.^1^, ^2^, ^3^ Shortly thereafter, it was shown that the disease was caused by a novel beta coronavirus (CoV) subsequently called SARS‐CoV‐2,^4^ and the respiratory disease caused by this virus was named COVID‐19.^5^, ^6^ Similar to SARS‐CoV, SARS‐CoV‐2 binds with the receptor‐binding domain (RBD) of its spike (S) protein to and enters the target cells via the angiotensin converting enzyme 2 (ACE2) receptor;^7^, ^8^, ^9^ however, alternative receptors for cell entry such as CD147/EMMPRIN/Basigin^10^ and CD299 (formerly CD209L) may exist.^11^, ^12^ SARS‐CoV‐2 is highly infectious and is easily spread by aerosols.^13^, ^14^

Accordingly, since December 2019 and until September 15, 2020, SARS‐CoV‐2 has infected 29 281 638 people worldwide and caused 928 423 deaths, representing a mortality rate of 3.44%. Austria has been similarly affected by the pandemic, with 33 541 infected persons and 757 deaths to date (https://coronavirus.jhu.edu/map.html).

Current data indicate a long and relatively mild initial course of the disease after SARS‐CoV‐2 infection, which may be followed by a severe course after a median time of 8 days from the first symptoms. The mild period may be followed by a period of breathlessness (approximately on day 8), which may turn into acute respiratory distress syndrome (ARDS) on day 9, followed by admission to intensive care on day 10.5.^15^, ^16^, ^17^ Acute infection with SARS‐CoV‐2 can be proven by detecting nucleic acids from the virus in nasopharyngeal swabs or lavages.^18^ After the acute infection, SARS‐CoV‐2‐specific antibodies^9^, ^16^ are usually formed, which coincides with the clearance of the virus from the body and a delayed response might be associated with mortality.^19^ However, SARS‐CoV‐2‐specific antibodies have varying specificities and titers for different antigens/epitopes and it has been shown that antibodies from convalescents after a mild COVID‐19 infection cannot always prevent the virus from binding to ACE2 and may therefore not have a protective effect.^9^

Similarly, several studies have investigated the effect(s) of a primary SARS‐CoV‐2 infection on leukocyte populations in the body in general, and on T and B lymphocyte subsets in particular. Also, the SARS‐CoV‐2‐specific T cell response has been investigated by several research groups.^17^, ^29^

Notably, memory CD4^+^ T cells have been suggested to mediate protective immunity against the previously circulating CoV‐strains SARS‐CoV^30^ and MERS,^31^ respectively, and CD8^+^ memory T cell responses have been observed in SARS‐CoV convalescent patients years after primary infection.^32^ However, regarding infection with the novel coronavirus SARS‐CoV‐2 recent reports have revealed conflicting results with one study (analyzing PB lymphocyte subsets in 44 patients) pointing toward reduced T memory and T regulatory cell populations in severe COVID‐19‐infected patients, suggesting that immune dysregulation potentially leads to aggravated inflammatory responses in patients.^28^ In contrast, another study provided evidence for segregation between memory T cells from patients with acute severe or acute moderate COVID‐19 disease and the development of memory T cells from recently convalescent individuals (42‐58 days after infection) and healthy controls.^33^ The latter study found only very moderate changes in a group of 40 COVID‐19 convalescent patients who underwent a mild COVID‐19 disease course (49‐64 days after disease onset).

However, the available studies on cellular immune responses were mainly conducted in patients suffering from acute COVID‐19 but little is known about the protracted effects of COVID‐19 on cellular immune responses. Consequently, there is a need for systematic studies analyzing COVID‐19 effects on cellular immune responses in cohorts of well documented COVID‐19 convalescent patients, in particular, for those who had experienced mild disease constituting the majority of cases, to understand if and how the infection continues to stimulate and/or perturb the immune system of the convalescent host. It is also unclear how possible changes in peripheral leukocyte/lymphocyte compartments correlate with SARS‐CoV‐2‐specific antibody levels and/or clinical symptoms.

Accordingly, the aims of this study were to identify the immunological imprint of primary COVID‐19 infection on peripheral blood (PB) cell populations and antibody levels. We were especially interested whether distinct changes in blood cell or antibody parameters would separate COVID‐19 convalescent patients from healthy subjects and whether we could define parameters which may be associated with the disease course, disease duration, pre‐existing medical conditions, regular medications, or demographic parameters. Thus, we performed here a systematic study to investigate the distribution of leukocyte subsets in peripheral blood with a special interest in T and B lymphocyte subpopulations of COVID‐19 convalescent patients 10 weeks after mild SARS‐CoV‐2 infection and compared results with a large, age‐matched, healthy control group, which was recruited in parallel and was negative for SARS‐CoV‐2 antibodies and had a negative SARS‐CoV‐2 rtPCR at the time of venipuncture. Therefore, and in order to unequivocally determine leukocyte, T and B lymphocyte populations we performed whole‐blood multiparametric flow cytometry on COVID‐19‐infected patient and control peripheral blood (PB) samples. Furthermore, we determined SARS‐CoV‐2‐specific antibody levels against S protein, its subunit the RBD protein, and the viral nucleocapsid protein and analyzed whether thymic and/or bone marrow output were affected in COVID‐19‐infected patients who underwent a mild disease course.

## 2 MATERIALS AND METHODS

### 2.1 Patients, control subjects, and trial conduct

Between May 11, 2020, and July 2, 2020, 109 patients diagnosed with COVID‐19 disease 10 weeks previously (71.2 ± 16.5 days) were enrolled into this study. The 109 patients had rtPCR‐confirmed (n = 92, 84.4%) and/or SARS‐CoV‐2 antibody‐confirmed (Elecsys^®^ Anti‐SARS‐CoV‐2 assay Roche) (n = 108 tested, n = 107 positive, 99.1%) COVID‐19 disease.^34^ In parallel, 98 healthy control subjects, who were reportedly asymptomatic for the last 10 weeks and who were SARS‐CoV‐2 negative by certified SARS‐CoV‐2 antibody test (Elecsys^®^ Anti‐SARS‐CoV‐2 assay Roche) and had a negative rtPCR test for SARS‐CoV‐2 at the time of venipuncture, were enrolled into the study. Similar to the COVID‐19‐infected patients also the HC collective had different co‐morbidities as specified in Table 1. All patients gave their written informed consent in accordance with the Declaration of Helsinki. The study was approved by the Ethics Committee of the Medical University of Vienna (EK No.: 1302/2020). Venous blood was drawn from all subjects and was either EDTA‐anticoagulated (for flow cytometric analyses and determination of lymphocyte emigration rates from thymus using TREC and bone marrow using KREC technology), heparin‐anticoagulated (for cryopreservation of PBMC), or silicon dioxide coagulated (to obtain serum for determining specific antibodies).

**Table 1:** Demographics, clinical presentation, and pre‐existing health conditions of COVID‐19 convalescent patients and healthy control subjects

Characteristics no. (%)	COVID‐19 convalescent patients N = 109	Healthy control subjects N = 98
Female sex	48 (44.0)	54 (55.1)
Age Median (Range) (Mean ± SD)	52 (16‐78) 50.1 ± 14.1	51 (14‐77) 50.1 ± 14.2
Height in cm (Mean ± SD)	175.6 ± 8.9	172.2 ± 8.9
Weight in kg (Mean ± SD)	78.1 ± 16.9	73.7 ± 16.4
Positive PCR COVID‐19 test	92 (84.4)	0 (0)
Positive antibody test (Roche)	107 (99.1)	0 (0)
Sneeze	27 (24.8)	0 (0)
Mild	21 (19.3)	0 (0)
Moderate	5 (4.6)	0 (0)
Strong	1 (0.9)	0 (0)
Runny nose	32 (29.4)	0 (0)
Mild	21 (19.3)	0 (0)
Moderate	9 (8.3)	0 (0)
Strong	2 (1.8)	0 (0)
Nasal congestion	47 (43.1)	0 (0)
Mild	29(26.6)	0 (0)
Moderate	11 (10.1)	0 (0)
Strong	7 (6.4)	0 (0)
Conjunctivitis	13 (11.9)	0 (0)
Mild	9 (8.3)	0 (0)
Moderate	3 (2.8)	0 (0)
Strong	1 (0.9)	0 (0)
Arthralgia	55 (50.5)	0 (0)
Mild	18 (16.5)	0 (0)
Moderate	16 (14.7)	0 (0)
Strong	21 (19.3)	0 (0)
Myalgia	56 (51.4)	0 (0)
Mild	18 (16.5)	0 (0)
Moderate	12 (11.0)	0 (0)
Strong	26 (23.9)	0 (0)
Nausea	22 (20.2)	0 (0)
Mild	14 (12.8)	0 (0)
Moderate	5 (4.6)	0 (0)
Strong	3 (2.8)	0 (0)
Headache	72 (66.1)	0 (0)
Mild	19 (17.4)	0 (0)
Moderate	22 (20.2)	0 (0)
Strong	31 (28.4)	0 (0)
Chills or rigor	54 (49.5)	0 (0)
Mild	23 (21.1)	0 (0)
Moderate	18 (16.5)	0 (0)
Strong	13 (11.9)	0 (0)
Fever	84 (77.1)	0 (0)
Max. temperature in °C Median (Range) Mean ± SD	38.5 (37.1‐42.0) 38.5 ± 0.8	n.a.
<37.5°C	2 (1.8)	n.a.
37.5‐38.0°C	23 (21.1)	n.a.
38.1‐39.0°C	43 (39.4)	n.a.
>39.0°C	17 (15.6)	n.a.
Fever duration in days (Mean ± SD)	4.3 ± 4.8	n.a.
Diarrhea	30 (27.5)	0 (0)
Mild	19 (17.4)	0 (0)
Moderate	10 (9.2)	0 (0)
Strong	1 (0.9)	0 (0)
Loss of taste/smell	72 (66.1)	0 (0)
Mild	19 (17.4)	0 (0)
Moderate	15 (13.8)	0 (0)
Strong	38 (34.9)	0 (0)
Fatigue	92 (84.4)	0 (0)
Mild	28 (25.7)	0 (0)
Moderate	19 (17.4)	0 (0)
Strong	45 (41.3)	0 (0)
Vomiting	7 (6.4)	0 (0)
Mild	7 (6.4)	0 (0)
Moderate	0 (0)	0 (0)
Strong	0 (0)	0 (0)
Other GI problems	7 (6.4)	0 (0)
Sore throat	42 (38.5)	0 (0)
Mild	27 (24.8)	0 (0)
Moderate	13 (11.9)	0 (0)
Strong	2 (1.8)	0 (0)
Cough	76 (69.7)	0 (0)
Mild	35 (32.1)	0 (0)
Moderate	20 (18.3)	0 (0)
Strong	21 (19.3)	0 (0)
Wheezing	26 (23.9)	0 (0)
Mild	11 (10.1)	0 (0)
Moderate	11 (10.1)	0 (0)
Strong	4 (3.7)	0 (0)
Shortness of breath	46 (42.2)	0 (0)
Mild	19 (17.4)	0 (0)
Moderate	18 (16.5)	0 (0)
Strong	9 (8.3)	0 (0)
Pneumonia	5 (4.6)	0 (0)
Mild	4 (3.7)	0 (0)
Moderate	1 (0.9)	0 (0)
Strong	0 (0)	0 (0)
Days of illness (Symptoms)	**15.5 ± 13.5**	**n.a.**
Symptom‐free days to venipuncture	55.3 ± 18.0	>70
Days between start of disease and venipuncture	71.2 ± 16.5	n.a
Hospitalized	8 (7.3)	0 (0)
Days of hospitalization	5.9 ± 5.8	0 (0)
ICU or IMCU admission	0 (0)	0 (0)
Days spent in ICU	0 (0)	0 (0)
O2 therapy required	3 (2.75)	0 (0)
Days of O2 therapy	3.0 ± 3.0	0 (0)
Invasive ventilation	0 (0)	0 (0)
Days of invasive ventilation	0 (0)	0 (0)
Asymptomatic	3 (2.8)	98 (100)
Pre‐existing health conditions	71 (65.1)	64 (65.3)
Cardiovascular diseases	22 (20.2)	19 (19.4)
Chronic lung diseases	15 (13.8)	11 (11.2)
Allergy/Asthma	38 (34.8)	43 (43.9)
Diabetes mellitus	3 (2.8)	4 (4.1)
Hematopoietic diseases	3 (2.8)	0 (0)
Immunosuppressive conditions	7 (6.4)	2 (2.0)
Liver diseases	2 (1.8)	3 (3.1)
Metabolic diseases	21 (19.3)	17 (17.3)
Neurological disorders	9 (8.3)	8 (8.1)
Renal diseases	4 (3.7)	3 (3.1)
Other medical problems	21 (19.3)	16 (16.3)
Pregnancy	0 (0)	0 (0)
Smoking	7 (6.4)	19 (19.4)
Regular alcohol consumption	41 (37.6)	36 (36.7)
Regular medications	49 (45.0)	48 (49.0)

### 2.2 Immunophenotyping by multiparametric flow cytometry

Immunophenotyping was performed by using optimal concentrations of directly conjugated monoclonal antibodies (Table S1) to leukocyte and lymphocyte (sub)populations according to standard quality controlled (inter‐laboratory test validated) procedures.^35^

Additional methods can be found in the Appendix 1 in the online repository to this article.

## 3 RESULTS

### 3.1 Clinical characterization of COVID‐19 convalescent patients

Between May 11, 2020, and July 2, 2020, 109 COVID‐19 convalescent patients and 98 healthy control subjects were enrolled into this retrospective study. The group of COVID‐19 convalescent patients and the group of healthy control subjects were well balanced regarding gender, age, height, and weight (Table 1). COVID‐19 convalescent patients were studied ~10 weeks after disease and healthy control subjects had no symptoms indicative of COVID‐19 or a common cold 10 weeks before participating in the study. COVID‐19 convalescent patients analyzed in this study (n = 109) consisted of 61 males and 48 females with a median age of 52 years (range 16‐78), and a mean COVID‐19 disease duration of 15.5 ± 13.5 days (range, 0‐66 days). The COVID‐19 disease symptoms in convalescent patients started 10 weeks (71.2 ± 16.5 (mean ± SD) days) before venipuncture/enrollment into this study. Seventy‐seven percent of patients presented with elevated body temperature (Table 1) for 4.3 ± 4.8 days with a median body temperature of 38.5°C (range 37.1‐42.0), 55% of patients had fever (>38°C). The most frequent clinical symptoms (Table 1) were fatigue (84.4%), followed by cough (69.7%), loss of taste or smell (66.1%), headache (66.1%), myalgia (51.4%), arthralgia (50.5%), chills or rigor (49.5%), nasal congestion (43.1%), shortness of breath (42.2%), and sore throat (38.5%). Gastrointestinal symptoms were much less frequently observed with diarrhea (27.5%), nausea (20.2%), and vomiting (6.4%). Of the 109 COVID‐19‐infected patients in this cohort, only eight (7.3%) had to be hospitalized for a mean of 5.9 ± 5.8 days, with three patients requiring noninvasive O_2_‐therapy. At the time of venipuncture, patients were symptom free for 55.3 ± 18.0 days. Seventy‐one patients (65.1%) had a pre‐existing health condition, while forty‐nine patients (45%) took regular medications (Table S2). None of the patients was pregnant, 6.4% of patients reported to be smokers and 37.6% of patients reported to drink alcohol on a recreational basis.

The control group consisted of healthy subjects (Table 1), who did not have COVID‐19‐associated disease symptoms for the last 70 days before venipuncture. Control subjects were enrolled and analyzed in parallel to the COVID‐19 convalescent patients and consisted of 98 individuals, 44 males (44.9%) and 54 females (55.1%) with a median age of 51 years (range 14‐77). Per definition, all subjects within the control group had a negative rtPCR test at the time of venipuncture and all tested negative with a certified commercial SARS‐CoV‐2 antibody test (Elecsys® Anti‐SARS‐CoV‐2 assay, Roche).

### 3.2 Primary SARS‐CoV‐2 infection induces protracted reduction of neutrophil counts and reveals increased numbers of activated cytotoxic T cells in the circulation

**Figure 1:**
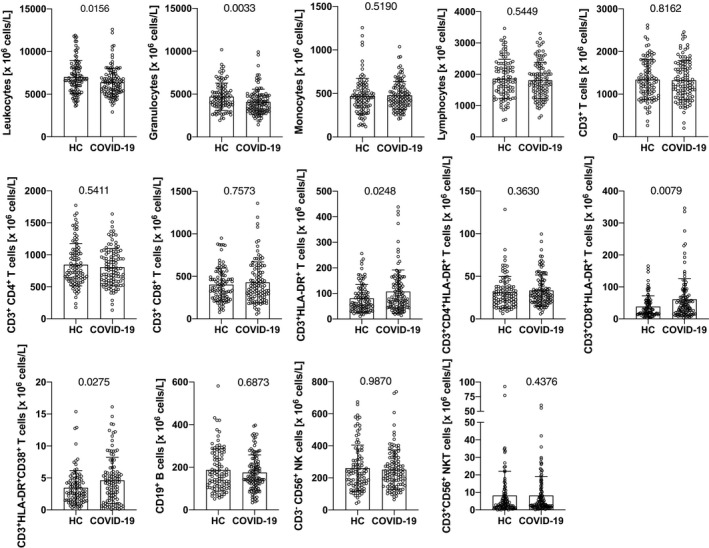
Impact of primary SARS‐CoV‐2 infection on absolute counts of leukocyte subpopulations as determined 10 weeks after disease onset. Shown are absolute values of the indicated leukocyte populations in peripheral blood of healthy control subjects (HC) and COVID‐19 convalescent patients (COVID‐19). Bars show mean values, whiskers the standard deviation, and open circles the values of single individuals. Data show pooled results of daily stainings (whole blood, 5‐9 individuals per day) of n = 98 for HC, and n = 109 for COVID‐19 convalescent patients, except for panels determining HLA‐DR expression where n = 97 for HC. P‐values were determined by Mann‐Whitney U‐test and are indicated

First, we determined overall peripheral blood (PB) leukocyte subpopulations in the COVID‐19‐infected patient collective and in healthy control subjects. Remarkably, the COVID‐19 convalescent patients showed significantly less leukocytes compared with the healthy control subjects (6384 ± 1677 × 10^6^/L vs 7006 ± 1961 × 10^6^/L; *P* = .0156), which was due to highly significantly lower neutrophil counts (4111 ± 1477 × 10^6^/L vs 4693 ± 1588 × 10^6^/L; *P* = .0033) (Figure 1; Figure S1 and Table S3). Neutropenia is frequently associated with trivial acute viral infections, but usually resolves after one week.^36^ While all COVID‐19‐infected patients studied herein had rtPCR‐ and/or anti‐SARS‐CoV‐2‐confirmed COVID‐19 disease, possible co‐infections with other viruses at the time of COVID‐19 infection cannot be excluded. Due to the enormous case‐load of PCR tests in the period March‐May, that is, when the first wave of the COVID‐19 pandemic was at its peak in Austria, no capacity for additional PCR tests was available, which represents a limitation of this study.

In contrast to neutrophils, numbers of monocytes, CD3^+^ T cells, irrespective whether TCR alpha/beta^+^ or TCR gamma/delta^+^, similar to the CD3^+^CD4^+^ and CD3^+^CD8^+^ T‐cell subsets, CD56^+^ NK cells and CD19^+^ B cells did not reveal significant differences between COVID‐19 convalescent patients and healthy control individuals (Figure 1 and Table S3). However, CD3^+^ T cells of COVID‐19 convalescent patients showed distinct signs of activation, with significantly more CD3^+^ T cells expressing HLA‐DR on their surface (107 ± 85 × 10^6^/L vs 81 ± 54 × 10^6^/L, *P* = .0248; 5.78 ± 3.84% vs 4.34 ± 2.50%; *P* = .0054) (Table S3). That HLA‐DR is a well‐known activation antigen of human T lymphocytes^37^ which is also associated with acute viral infections^38^, ^39^ has been described previously. T‐cell subset analyses revealed that the subset of cytotoxic CD3^+^CD8^+^ T cells exclusively accounted for that difference (61 ± 65 × 10^6^/L vs 38 ± 34 × 10^6^/L; *P* = .0079; 3.21 ± 2.91% vs 2.05 ± 1.61%; *P* = .0014), while CD3^+^CD4^+^ T cells had no increased signs of activation when compared for HLA‐DR expression to those of healthy controls (33 ± 18 × 10^6^/L vs 31 ± 19 × 10^6^/L; *P* = .3630) (Figure 1 and Figure S2). Activation of the T‐cell compartment in COVID‐19 convalescent patients was also evident when we examined CD38 co‐expression on CD3^+^ T cells (4.6 ± 3.6 × 10^6^/L vs 3.5 ± 2.7 × 10^6^/L; *P* = .0275; 0.27 ± 0.24% vs 0.19 ± 0.14%, *P* = .0051), which is another important activation antigen and also an endothelial adhesion molecule known for its upregulation on virus‐specific T cells.^38^, ^39^ Notably, and in clear contrast to HLA‐DR expression which was restricted to CD3^+^CD8^+^ T cells, significant upregulation of CD38 on lymphocytes of COVID‐19 convalescent patients was observed on both, CD4^+^ and CD8^+^ T cells (Table S3).

### 3.3 Numbers of naïve and effector memory CD3^+^CD4^+^ T helper cells and effector memory CD3^+^CD8^+^ cytotoxic T cells are higher in the blood of COVID‐19 convalescent patients compared with healthy controls

**Figure 2:**
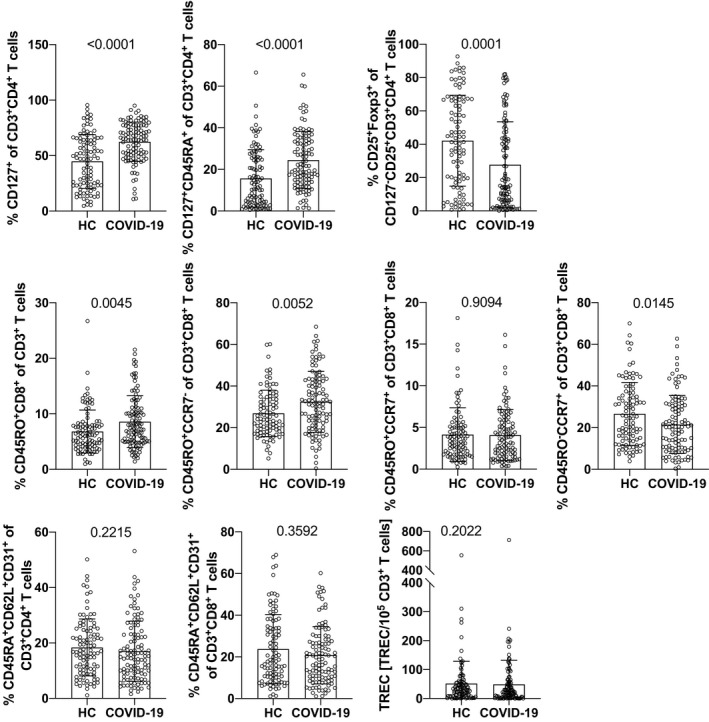
Impact of primary SARS‐CoV‐2 infection on relative amounts of T cell subpopulations and TREC levels as determined 10 weeks after disease onset. Shown are the relative values of selected T cell subpopulations in PB of healthy control subjects (HC) and COVID‐19 convalescent patients (COVID‐19). Bars show mean values, whiskers the standard deviation, and open circles the values of single individuals. Data show pooled results of daily stainings (whole blood, 5‐9 individuals per day) and TREC analyses of n = 98 for HC, and n = 109 for COVID‐19 convalescent patients, except for panels determining CD25/Foxp3 n = 97 for HC; and CD45RO/CCR7 n = 95 for HC and n = 105 for COVID‐19. P‐values were determined by Mann‐Whitney U‐test and are indicated. TREC, T cell receptor excision circles

Notably, a dramatic increase of the subset of CD3^+^CD4^+^ T cells co‐expressing the high affinity IL‐7 receptor alpha chain (CD127) irrespective of the presence (137 ± 114 × 10^6^/L vs 79 ± 106 × 10^6^/L, *P* < .0001; 24.47 ± 13.68% vs 15.66 ± 13.79%, *P* < .0001) or absence (498 ± 226 × 10^6^/L vs 370 ± 248 × 10^6^/L; *P* < .0001; 62.44 ± 17.69% vs 44.83 ± 24.22%, *P* < .0001) of CD45RA co‐expression and marking naïve and effector memory CD3^+^CD4^+^ T helper cells, respectively, was found (Figure 2; Figures S3, S4A and Table S4). This dramatic expansion appeared to be at the expense of Foxp3^+^CD25^+^CD127^‐^CD3^+^CD4^+^ T regulatory cells, which were significantly reduced in patients compared with healthy controls (11 ± 15 × 10^6^/L vs 15 ± 11 × 10^6^/L, *P* = .0004; 27.73 ± 25.70% vs 42.10 ± 27.26%, *P* = .0001) (Table S4).

Apart from the clear‐cut expansion of naïve and effector CD4^+^ T helper cells, significantly more CD3^+^CD8^+^CD45RO^+^CD45RA^‐^ memory T cells were present in the blood of COVID‐19 convalescent patients as compared to healthy controls (113 ± 79 × 10^6^/L vs 87 ± 54 × 10^6^/L; *P* = .0263) (Figure 2, Figure S4B), which was also reflected by their significantly higher relative levels among CD3^+^ T cells (8.58 ± 4.67% vs 6.82 ± 3.88%; *P* < .0045) (Table S4). The elevated levels of memory T cells almost exclusively belonged to the CD45RO^+^CCR7^‐^ effector memory subset (128 ± 89 × 10^6^/L vs 99 ± 55 × 10^6^/L; *P* = .0502), which was significantly higher among CD3^+^CD8^+^ T cells (32.42 ± 14.65% vs 26.90 ± 11.11%; *P* = .0052), while this was not evident for the central memory subset of CD3^+^CD8^+^CD45RO^+^CCR7^+^ (15 ± 14 vs 15 ± 10 10^6^/L; *P* = .7532) (Figure 2; Figures S3). Although CD3^+^CD8^+^ effector memory cells mostly belonged to the subset of early effector cells as defined by their co‐expression of CD27 and CD28,^40^ COVID‐19 convalescent patients had a significantly lower frequency of this subset among their CD3^+^CD8^+^CD45RO^+^CCR7^‐^ effector memory T cells (65.6 ± 13.0 vs 69.7 ± 13.0%, *P* = .0129), which was at the expense of more differentiated CD3^+^CD8^+^ T effector memory subsets, that is, those co‐expressing CD27^+^CD28^‐^ and CD27^‐^CD28^‐^, respectively (Figure S5). Notably, higher numbers of CD3^+^CD8^+^ memory T cells were paralleled by a decrease in absolute (85 ± 66 × 10^6^/L vs 108 ± 92 × 10^6^/L; *P* = .0704) and relative (21.52 ± 13.91% vs 26.60 ± 15.13%; *P* = .0145) numbers of CD3^+^CD8^+^CD45RO^‐^CCR7^+^ naïve cytotoxic T cells.

Moreover, moderately lower numbers of CD3^+^CD4^+^CD45RA^+^CD62L^+^CD31^+^ recent thymic emigrant (RTE) cells (141 ± 103 vs 165 ± 126; *P* = .2078; 17.01 ± 10.85% vs 18.40 ± 10.27%, *P* = .2215) were observed. Similarly, relative (20.81 ± 13.78% vs 23.73 ± 16.64%; *P* = .3592) and absolute (86 ± 70 × 10^6^/L vs 100 ± 95 × 10^6^/L; *P* = .5770) amounts of CD3^+^CD8^+^CD45RA^+^CD62L^+^CD31^+^ recent thymic emigrant T cells were lower; however, both changes did not reach statistical significance. The above findings may be indicative of decreased thymic output of both CD4^+^ and CD8^+^ T cells. However, determination of T cell receptor excision circles of PB T cells (TRECs), which negatively correlated with the increased age of our patients and correlated with the numbers of both CD3^+^CD4^+^ and CD3^+^CD8^+^ RTE did not indicate problems with thymic output in COVID‐19 convalescent patients with a mild disease course when compared to healthy control subjects (Figure 2; Figures S3).

### 3.4 Levels of transitional B cells and plasmablasts are higher in the blood of COVID‐19 convalescent patients while B memory compartments are unaffected

**Figure 3:**
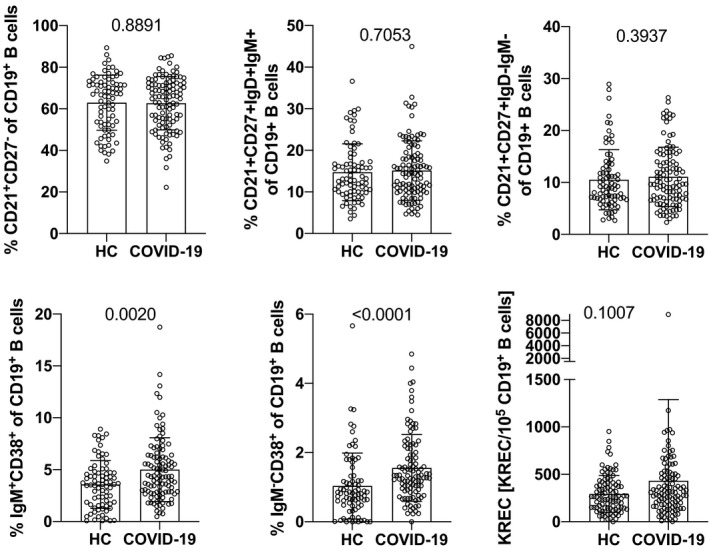
Impact of primary SARS‐CoV‐2 infection on relative amounts of Bcell subpopulations and KREC levels as determined 10 weeks after disease onset. Shown are the relative values of the indicated Bcell subpopulations in PB of healthy control subjects (HC) and COVID‐19 convalescent patients (COVID‐19). Bars show mean values, whiskers the standard deviation, and open circles the values of single individuals. Data show pooled results of daily stainings (whole blood, 5‐9 individuals per day of n = 78 for HC, and n = 108 for COVID‐19 convalescent patients)and KREC analyses (n = 98 for HC and n = 109 for COVID‐19). P‐values were determined by Mann‐Whitney U‐test and are indicated. KREC, kappa‐deleting recombination excision circles

Next, we determined B‐cell subset distribution and found that major B‐cell subpopulations such as naïve B cells, non–class‐switched, and class‐switched memory B cells were not different between COVID‐19 convalescent patients and healthy control subjects (Figure 3; Figures S6, S7 and Table S5). This finding was interesting, given the observed and above‐described gross distortions of T memory cell populations in COVID‐19 convalescent patients. However, CD19^+^CD38^high^IgM^+^ transitional B cells were significantly higher in COVID‐19 convalescent patients when compared to healthy control subjects (5.00 ± 3.07% vs 3.57  ± 2.30%; *P* = .0020) (Figure 3; Figures S6, S7 and Table S5). Similarly, CD19^+^CD38^+^IgM^‐^ plasmablasts were both absolutely (2.5 ± 1.8 × 10^6^/L vs 1.6 ± 1.3 × 10^6^/L; *P* = .0015) and relatively (1.56 ± 0.97% vs 1.04 ± 0.95%; *P* < .0001 higher in COVID‐19‐infected patients as compared to healthy control subjects (Figure 3; Figures S6, S7 and Table S5). That the current bone marrow output of B‐cell precursors was not depressed in COVID‐19 convalescent patients could be demonstrated by determining kappa recombination excision circles (KRECs), which were not different from the healthy control population (Figure 3).

### 3.5 Multivariate logistic regression analyses confirm associations between COVID‐19 disease and imprint on immune cell parameters

In a next step, multivariate logistic regression analyses were performed for the 46 significant blood cell and serum parameters identified in univariate analyses. Receiver operating characteristic (ROC) curves (Figure S8) demonstrate that COVID‐19 convalescent patients and HC subjects differ regarding several immune cell parameters. Indeed, elevated numbers of CD3^+^CD4^+^CD45RA^+^CD127^+^ naïve T helper cells (area under the curve (AUC) 66.2%; 95% confidence interval (CI) 58.1%‐74.3%; *P* < .001) and of cytotoxic CD3^+^CD8^+^CD45RO^+^CCR7^‐^ effector memory T cells (AUC 60.1%; 95% CI 51.8% to 68.5%; *P* = .022) were clearly higher in COVID‐19 convalescent individuals than HC. In contrast, relative numbers of CD3^+^CD4^+^CD45RA^+^Foxp3^+^CD25^+^CD127^‐^ T regulatory cells (*P* = .047) and of antigen‐experienced(AE)3 cytotoxic CD3^+^CD8^+^CD27^+^CD28^‐^CD45RA^+/‐^ T cells (*P* = .012) were significantly lower in COVID‐19 convalescent patients when compared to HC subjects. Moreover, both higher frequencies of transitional CD19^+^CD38^+^IgM^high^ B cells (AUC 65.4%; 95% CI 57.4% to 73.5%; *P* < .001) as well as CD19^+^CD38^+^IgM^‐^ plasmablasts (AUC 70.3%; 95% CI 62.3 to 78.2%; *P* < .001) significantly discriminated COVID‐19 convalescent individuals from HC.

### 3.6 Associations between clinical parameters and changes in blood cells and serum parameters

**Figure 4:**
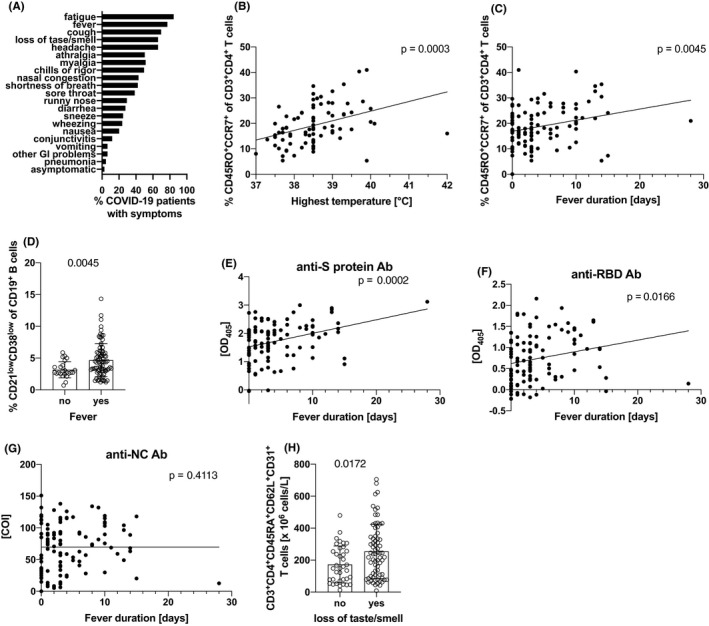
Clinical symptoms of COVID‐19‐infected patients and their correlation with cellular and humoral immune parameters. A, Shown are the frequencies of symptoms observed in COVID‐19‐infected patients (n = 109). B to H, Shown are the correlations of cellular (B to E) and humoral (F to H) immune parameters with typical COVID‐19 symptoms (n = 85 in B; n = 109 in C; n = 23 for no and n = 84 for fever in D; n = 109 for E, F and n = 108 for G; n = 37 for no and n = 72 for loss of taste/smell in H). In B, only patients presenting with fever are shown. Statistical significance was determined by Mann‐Whitney U‐test for categorical values and by Pearson's correlation for continuous values. Lines in B, C and F to H represent the trend. COI, cut‐off index

Next, we performed univariate analyses in order to investigate whether blood cell parameters and/or SARS‐CoV‐2‐specific antibody levels (S‐, RBD‐, and NC‐protein) would correlate with clinical symptoms (Figure 4A), co‐morbidities, and premedications of COVID‐19‐infected patients. Most notably, we found that convalescent patients who had more severe fever during their disease had significantly higher relative numbers of CD3^+^CD4^+^CD45RO^+^CCR7^+^ central memory helper T cells (Figure 4B) in their blood (*P* = .0003). In addition, relative numbers of CD3^+^CD4^+^CD45RO^+^CCR7^+^ central memory helper T cells were significantly associated with chills (*P* = .0030; data not shown), and with the number of fever days (*P* = .0045; Figure 4C). These findings were corroborated by significant inverse correlations observed between highest fever and relative and absolute numbers of naïve CD3^+^CD4^+^CD45RO^‐^CCR7^+^ T cells (*P* = .0025 and *P* = .0034, respectively; not shown). Notably, patients who had fever during their disease also presented with significantly more activated CD19^+^CD21^low^CD38^low^ B cells ^41^ (Figure 4D; *P* = .0045).

Moreover, the number of fever days and the presence of fever in general (not shown), correlated well with both the levels of anti‐S‐protein IgG (*P* = .0002) and anti‐RBD IgG (*P* = .0166) as determined by ELISA (Figure 4E, F). In contrast, anti‐NC antibodies only moderately correlated with fever/highest temperature (not shown) but not duration of fever (Figure 4G).

Another very interesting finding was the fact that patients suffering from loss of taste/smell had significantly more CD3^+^CD45RA^+^CD62L^+^CD31^+^RTEs in their blood (P = 0.0172) (Figure 4H). While CD3^+^CD45RA^+^CD62L^+^CD31^+^ recent thymic emigrant cells positively correlate with TREC numbers (*P* < .0001) and negatively with biological age (*P* < .0001), this association was only evident when the age was determined “immunologically,” that is, by multicolor flow cytometry but not upon determination of birth age. This finding may indicate that individuals with a “young(er) immune system” are more likely to suffer from loss of taste/smell when infected by SARS‐CoV‐2. That loss of taste/smell represents indeed a separate clinical factor in COVID‐19‐infected patients, which is clearly different from, for example, the fever/coughing/fatigue‐ or the arthralgia/myalgia‐factor was confirmed by principal component analysis (PCA), reducing clinical symptoms to factors best explaining the variance of variables, followed by rotated component matrix relation determination (Varimax‐Rotation) using the collected clinical symptom categories (Table 2). These analyses revealed seven factors defined by certain symptoms (factor 1: fever, chills, rigor, fatigue, cough; factor 2: running nose, nasal congestion, sore throat, sneeze; factor 3: arthralgia, myalgia; factor 4: conjunctivitis, other GI problems; factor 5: pneumonia, shortness of breath, wheezing; factor 6: vomiting, nausea, headache, diarrhea; factor 7: other symptoms, loss of taste, smell) with an explained proportion of variance of 65.4% (KMO = 0.728; Bartlett's test of sphericity *P* < .001) and identified loss of taste/smell as a clearly separate clinical disease entity.

**Table 2:** Varimax‐rotated factor structure for reported clinical symptoms explaining a large proportion of the heterogeneity of clinical symptoms in COVID‐19‐infected patients

Symptom category	Factors						
	F1	F2	F3	F4	F5	F6	F7
Fever	0.758		0.183				−0.160
Chills or rigor	0.739		0.116		0.107		0.184
Fatigue	0.694	0.230	0.241			0.194	0.263
Cough	0.558			0.177	0.471	−0.112	0.258
Running nose		0.770			−0.102		
Nasal congestion		0.731		0.208		0.157	0.219
Sore throat	0.165	0.626	0.218	−0.125	0.394	0.113	
Sneeze	0.347	0.518	−0.244	0.135		−0.233	−0.172
Arthralgia			0.888	0.123			
Myalgia	0.242		0.845	0.119			
Conjunctivitis		0.146		0.759			0.273
Other GI problems			0.160	0.724			0.113
Pneumonia				−0.194	0.808	−0.143	
Shortness of breath	0.407			0.376	0.576	0.110	
Wheezing	0.317			0.472	0.525	0.387	−0.152
Vomiting		−0.145			−0.128	0.700	
Nausea	0.131	0.218	0.248	0.423	0.187	0.496	−0.168
Headache	0.225	0.297	0.393	−0.148		0.487	0.228
Diarrhea		0.446		0.246	0.108	0.475	0.217
Other symptoms				0.228		−0.115	0,772
Loss of taste/smell	0.155	0.223				0.312	0.663
Percent variance	22.7	10.7	8.8	7.0	6.1	5.3	4.8

Note

The total amount of variance explained by the seven factors (F1‐F7) defined by certain symptoms (factor 1: fever, chills, rigor, fatigue, cough; factor 2: running nose, nasal congestion, sore throat, sneeze; factor 3: arthralgia, myalgia; factor 4: conjunctivitis, other GI problems; factor 5: pneumonia, shortness of breath, wheezing; factor 6: vomiting, nausea, headache, diarrhea; factor 7: other symptoms, loss of taste, smell) adds up to 65.4%. The Kaiser‐Meyer‐Olkin criterion of 0.728 shows a middling value and the Bartlett‐Test of sphericity is significant (*P* < .000). Varimax‐rotated factor loadings for the indicated groups of clinical symptoms. Loadings lower than 0.1 are omitted from the table. Gray background indicates grouped symptom (termed F1 to F7) displaying highest factor loadings.

Not unexpectedly, the age of patients correlated with the prevalence of cardiovascular, metabolic and renal diseases in the COVID‐19 convalescent patients, however, surprisingly not with disease duration and severity (fever days, fever height) although age is frequently associated with a higher mortality risk.^42^ Notably, a total of 45% of COVID‐19 convalescent patients took regular medications (Table S2) with 16 out of the 109 patients reporting intake of ACE‐ or angiotensin receptor (AT) blockers (candesartan, lisinopril, enalapril, ramipril, irbesartan, valsartan), that means, substances which have been shown to lead to increased ACE2 receptor expression in cardiac tissue in preclinical models previously.^43^ While ACE/AT inhibitor intake positively correlated with the age of patients (*P* < .0001), it apparently did not correlate with the disease course, for example, duration of fever (*P* = .7316) or of clinical symptoms (*P* = .6131). Of significance, also 11 subjects in the control group took ACE/AT inhibitors as regular medication; thus, there was no significant difference between the patient and control group regarding ACE/AT inhibitor consumption.

## 4 DISCUSSION

We here show that acute SARS‐CoV‐2 infection has protracted impacts on the human immune system even in COVID‐19 convalescent patients who underwent a mild disease course. Our data support the view that SARS‐CoV‐2 challenges the human immune system at different levels. We show that even 70 days after acute SARS‐CoV‐2 infection protracted reduction of neutrophil counts is observed in COVID‐19 convalescent patients (n = 109) when compared to healthy control subjects (n = 98), which is paralleled by activation of T cells as demonstrated by higher HLA‐DR (CD8^+^ T cells) and CD38 (CD4^+^ and CD8^+^) expression. Moreover, significantly greater numbers of CD3^+^CD4^+^CD127^+^CD45RA^‐^ effector memory T cells as well as of CD3^+^CD8^+^CD45RO^+^CCR7^‐^ effector memory T cells clearly distinguished COVID‐19 convalescent individuals from healthy control subjects. While both B memory cell populations (non–class‐switched and class‐switched) did not differ between COVID‐19 convalescent patients and healthy control subjects, numbers of early B cells characterized as CD19^+^IgM^+^CD38^+^ transitional B cells as well as plasmablasts were higher in convalescents as compared to healthy controls. Increased plasmablast levels had been reported in severe COVID‐19‐infected patients at the time of the disease previously.^22^ SARS‐CoV‐2‐specific antibody levels were detectable in all but one patient who had received chemotherapy. Interestingly, anti‐S and anti‐RBD antibody levels as well as CD3^+^CD8^+^CD45RO^+^CCR7^+^ central memory T cells, but not anti‐nucleocapsid antibodies, correlated with duration of fever.

Virus‐induced reduction of neutrophils is a common feature of childhood infections including respiratory syncytial virus (RSV), influenza A and B, and parvovirus. In most cases, neutropenia occurs during the first few days of viral illness and lasts for a maximum of eight days.^36^ However, some viruses have been shown to be associated with decreased neutrophil numbers and even neutropenia, after the acute phase of infection, such as EBV,^44^ Hepatitis A virus,^45^ measles,^46^ varicella,^47^ phlebotomus fever virus (Sicilian type),^48^ and rubella.^49^ For the latter two viral infections, reduced neutrophil counts are maintained for up to 6 weeks after initial infection. The mechanistic basis for the reduced neutrophil counts may be either complement‐fixing anti‐neutrophil antibodies or exhaustion of the neutrophil pool.

The observation that even 70 days after infection T cells are expressing HLA‐DR and CD38 speaks for their sustained SARS‐CoV‐2‐dependent activation. Indeed, HLA‐DR becomes upregulated upon T‐cell activation^37^ and has been demonstrated to be increased on CD8^+^ cytotoxic T cells of patients suffering from autoimmune diseases^50^ or acute and chronic viral infections such as HIV^38^, ^39^, ^51^ and also in the acute phase of COVID‐19.^22^, ^52^ What may be the function of increased HLA‐DR surface expression on CD8^+^ T cells? Notably, HLA‐DR^+^ cytotoxic T cells have been shown to possess all the requirements for processing and loading of antigens onto HLA‐DR molecules.^53^ Moreover, cytotoxic T cells are also proficient to express the canonical co‐stimulatory molecules CD86 and CD80, which are essential for the priming and activation of effector T cells.^53^ In addition, a previous report has shown that T cell–T‐cell synapses have a major role in the generation of protective CD8^+^ T‐cell memory since conjugated cells critically polarize each other toward the secretion of interferon‐gamma (IFN‐gamma).^54^

We here also found increased CD38 expression on both CD4^+^ and CD8^+^ T cells of COVID‐19 convalescent patients. In fact, CD38 (adenosine diphosphate (ADP) ribosyl cyclase) marks T cells with increased cytotoxic capability^38^, ^39^ and enhanced ability to produce cytokines.^55^ Furthermore, CD38 endows T cells with improved interaction with endothelial cells via CD31,^56^ which has been shown to lead to improved adhesion of lymphocytes to endothelium.^57^ Whether this also leads to enhanced extravasation of T cells is an open question, but it is tempting to speculate that CD38^+^ T cells might have an improved capability to target sites of inflammation.

Antibodies and T cells are the two main antigen‐specific effector systems for resolving viral infections. Notably, both T cell subsets contribute to viral clearance.^58^, ^59^ In fact, anti‐viral CD4^+^ T cell responses are important for optimal antibody and CD8^+^ T cell responses. Thus, it is not entirely unexpected that we found significantly elevated numbers of effector memory CD4^+^CD45RA^+^CD127^+^ T cells in COVID‐19 convalescent patients. What is, however, surprising is the level and degree of maintenance of these cells in patients with a mild COVID‐19 disease course, because we could easily identify their high numbers even without applying antigen‐specific detection methods such as tetramer staining. One mechanism for the sustained expansion of these effector cells seems to be the mitigation of T regulatory responses, which is reflected by significantly lower CD25^+^Foxp3^+^CD127^‐^ T cells in convalescents as compared to healthy controls (Figure 2 and Table S4).

Whether the CD4^+^ effector cells have mere helper function for the clonal expansion of the SARS‐CoV‐2‐specific B cells or may also exert cytotoxic cellular programs remains to be shown in future analyses. Nevertheless, besides the expanded CD4^+^ T cells, we also detected a significant expansion of the CD8^+^ effector memory pool. These cells are supposedly cytotoxic and well‐apt to attack and lyse SARS‐CoV‐2‐infected cells. They also contribute to the activated CD8^+^ T cell pool found elevated in convalescents, which co‐expresses HLA‐DR and CD38. While lymphopenia was a constant finding in severely ill patients with acute SARS‐CoV‐2 infection,^21^ lymphopenia was not detected in our convalescent patient collective. Notably, thymic involution has been shown in the past to be associated with increased levels in the thymus of distinct cytokines, among them also interleukin (IL)‐6, which itself represents a promising biomarker for severely ill COVID‐19‐infected patients^60^, ^61^ and qualified in the recent past as a potent target for immune intervention in severely ill COVID‐19‐infected patients using Tocilizumab.^62^ However, the relatively mild disease course of our COVID‐19 convalescent patients did neither reveal reduced overall lymphocyte counts nor reduced thymic output 70 days after acute infection.

Apart from elevated numbers of memory T cells, we found elevated numbers of circulating plasmablasts, which are commonly referred to as a sign of recent antigen contact ^63^ and acute COVID‐19.^22^ The elevated plasma cell precursor levels perfectly fit to the broad anti‐SARS‐CoV‐2 antibody response in COVID‐19 convalescents. In fact, all but one COVID‐19 convalescent patient had specific antibody responses (108 tested; 107 positive) in the certified SARS‐CoV‐2 antibody test (Elecsys^®^ Anti‐SARS‐CoV‐2 assay, Roche). Interestingly, one COVID‐19 convalescent patient was completely negative in the Roche test, although the patient was tested positive by rtPCR for SARS‐CoV‐2 during acute disease. A closer look at this discrepant test result (PCR‐test positive; NC‐test negative) revealed that this patient underwent chemotherapeutic treatment for metastatic cancer. This patient presented not only with a negative NC‐test value of 0.1 (cutoff level 1.0) but also had no specific anti‐S or anti‐RBD antibodies levels. The finding that individuals with a suppressed immune system may well be able to cope with COVID‐19 and clear the virus without a clear sign of seroconversion is interesting because it indicates that virus clearance and disease resolution are eventually not only dependent on antibody responses.

Moreover, two patients clearly reacted in the NC‐test (36.0 and 74.1, respectively), but no anti‐S‐protein or anti‐RBD protein antibodies were detected. Together, these findings indicate that the spectrum and magnitude of antibody specificities in COVID‐19 convalescent patients may considerably vary. In this context, it will be interesting to perform a comprehensive analysis of the SARS‐CoV‐2‐specific antibody response using a broad spectrum of virus‐derived antigens and epitopes and testing of several isotypes and IgG subclasses by multiplex tests such as chips containing micro‐arrayed components similar as developed for other viral infections.^64^, ^65^

The fact that more than 25% of the convalescent patients did not mount a relevant anti‐RBD antibody response fits to our earlier finding that up to 50% of convalescent patients do not mount antibodies which can inhibit the docking of the virus via RBD to its receptor, ACE2.^9^ Also in this study, we found that ~50%‐60% of COVID‐19 convalescent patients have antibodies inhibiting the binding of RBD to ACE2 and hence may be considered to have neutralizing capacity, which confirmed the findings of our last study^9^ (data not shown).

Whether antibodies binding to S‐protein outside of the RBD have virus‐neutralizing capacity or contribute to virus clearance by other mechanisms such as receptor‐mediated clearance by immune cells remains to be investigated.

The percentages of pre‐existing allergy/asthma in both groups were quite high (COVID‐19 group: 34.8%; Control group: 43.9%), however, within the range reported for the general population in Austria.^66^ Within the COVID‐19‐infected patient group, no significant associations between allergy and the self‐reported severity of typical COVID‐19 symptoms such as coughing (*P* = .8413), shortness of breath (*P* = .2906), or wheezing (*P* = .0647) were evident.

In summary, we provide compelling evidence for a considerable and protracted imprint of SARS‐CoV‐2 infection on the human immune system at different levels even after mild COVID‐19 disease course. It is a limitation of our study that we have no data whether the COVID‐19‐infected patients besides SARS‐CoV‐2 had also other infections. However, their disease course was mild. Furthermore, the control subjects were from the same region and it is therefore likely that such other infections have affected both groups in a comparable manner. The observed differences between the COVID‐19 and control group are therefore most likely caused by the SARS‐CoV‐2 infection. Specifically, SARS‐CoV‐2 infection seems to leave beneficial (ie, activation of T cells, increased numbers of plasmablasts) and potentially harmful (ie, reduction of neutrophils, reduction of Tregs) imprints in the cellular immune system in addition to the induction of specific antibody responses. Several aspects of our study are of potential clinical relevance: (i) long fever duration was associated with increased T cell memory, that is, central memory CD4^+^ T help, which is in line with previous studies^67^ and higher anti‐S and anti‐RBD but not anti‐NC antibody levels, potentially implicating that such COVID‐19 convalescent individuals may have a better protection upon re‐exposure to SARS‐CoV‐2; (ii) however, patients unable to mount detectable anti‐SARS‐CoV‐2 antibody levels may still be able to efficiently clear the virus and recover from COVID‐19 diseases because we found one patient who had chemotherapy and, although this subject did not produce detectable SARS‐CoV‐2 antibodies recovered from COVID‐19; (iii) the reduced neutrophil numbers long after COVID‐19 disease are stunning and warrant the search for possible underlying mechanisms (eg, anti‐neutrophil antibodies); (iv) the dramatically low numbers of CD4^+^ Treg cells requires further investigation to clarify whether this is a potentially beneficial or harmful condition. The reduced CD4^+^ Tregs may facilitate anti‐SARS‐CoV‐2 immunity but the low CD4^+^ T regs may eventually foster auto‐reactivity; (v) the long‐term prevalence of HLA‐DR‐ and CD38‐ expressing T cells may be a sign of robust SARS‐CoV‐2‐specific immunity but may be also caused by persistence of antigen; and (vi) a more juvenile immune system, as determined by the numbers of RTE was associated with loss of sense of smell/taste as the main clinical symptom. It remains to be investigated whether there is a causal relationship between these findings.

Our study thus not only reports definitive evidence for a protracted immunological imprint of COVID‐19 on human peripheral leukocyte populations but also raises several new aspects of COVID‐19 which require further studies.

## CONFLICT OF INTEREST

With regard to the authors’ disclosure of potential conflicts of interest, we would like to indicate that Winfried F. Pickl holds stocks of Biomay AG and has received honoraria from Novartis, Astra Zeneca, and Roche. Rudolf Valenta has received research grants from HVD Life‐Sciences, Vienna, Austria, and from Viravaxx, Vienna, Austria. He serves as consultant for Viravaxx. The other authors have no conflict of interest to declare. Rainer Henning is an employee of Viravaxx, Vienna, Austria.

## AUTHOR CONTRIBUTIONS

B. K., R. V., and W. F. P. designed research; B. K., D. T., P. E., U. K., A. R., F. T., M. F., P. G., K. B., Y. D., I. T., M. W., K. G.‐P., P. A. T., M. G., B. M., T. P., I. F., S. W., H. F., R. H., R. V., and W. F. P. performed research and analyzed data; B. K., R. V., and W. F. P. wrote the paper. All authors critically read the paper and approved the manuscript.

## Supporting information

Fig S1

Fig S2

Fig S3

Fig S4

Fig S5

Fig S6

Fig S7

Fig S8

Supplementary Material
