# Supplementary figures and images for: Immunological imprint of COVID‐19 on human peripheral blood leukocyte populations
Source: Allergy. 2020 Nov 22;76(3):751–65. doi: 10.1111/all.14647 (PMC7984452; doi:10.1111/all.14647)

FIGURE S1

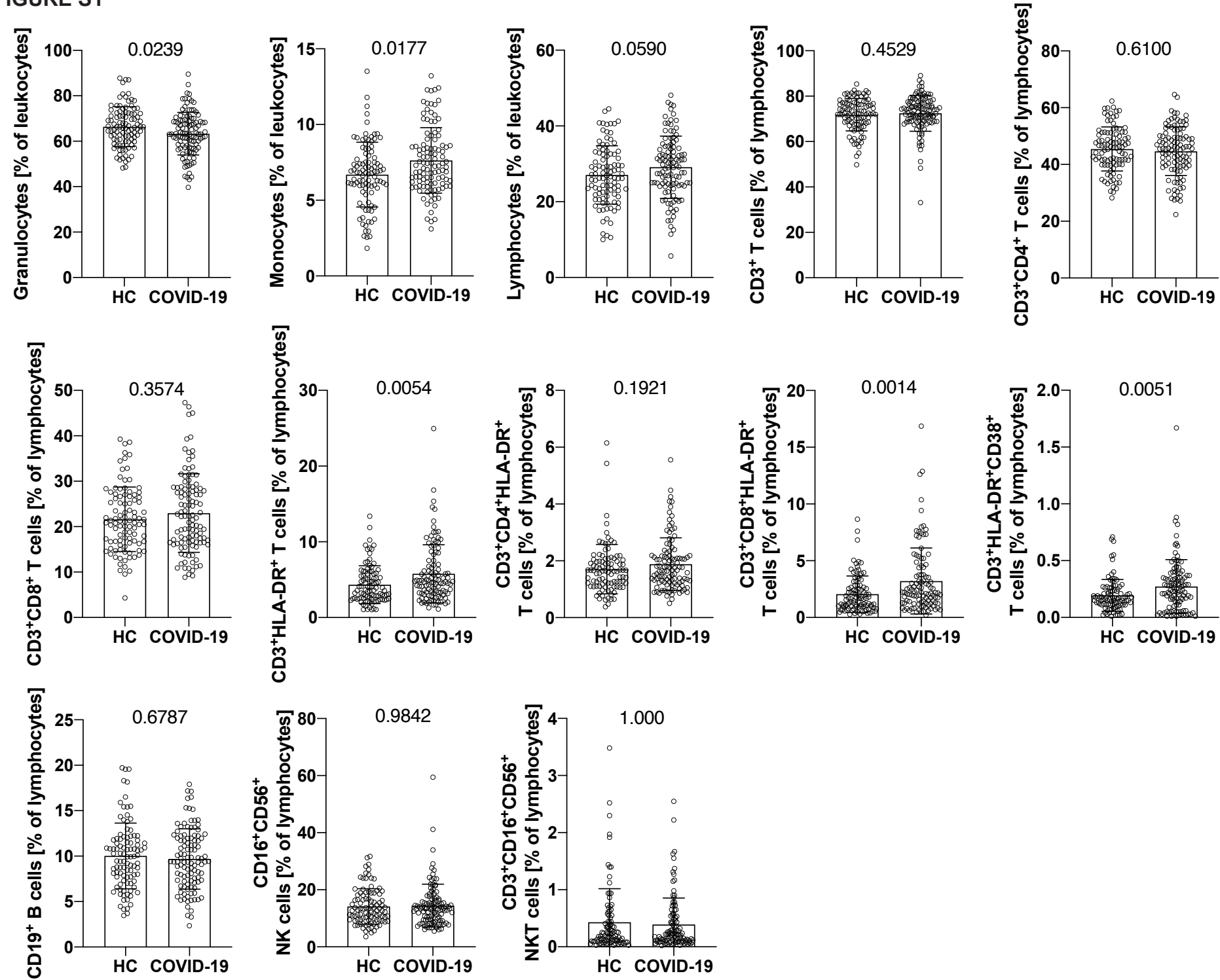

Supplement: Supplementary file 1 — Fig S1 [file ALL-76-751-s008.pdf]

FIGURE S2

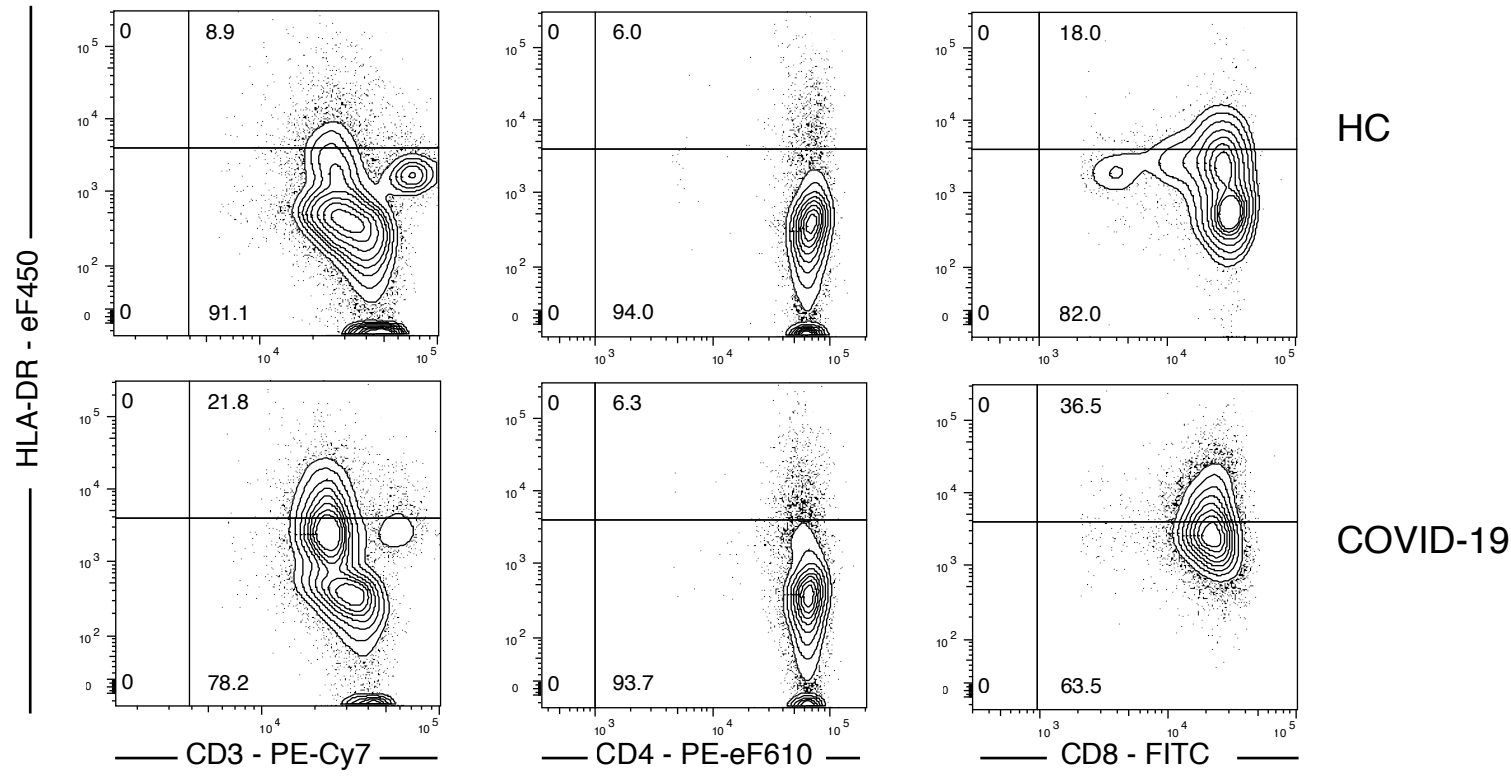

Supplement: Supplementary file 2 — Fig S2 [file ALL-76-751-s007.pdf]

FIGURE S3

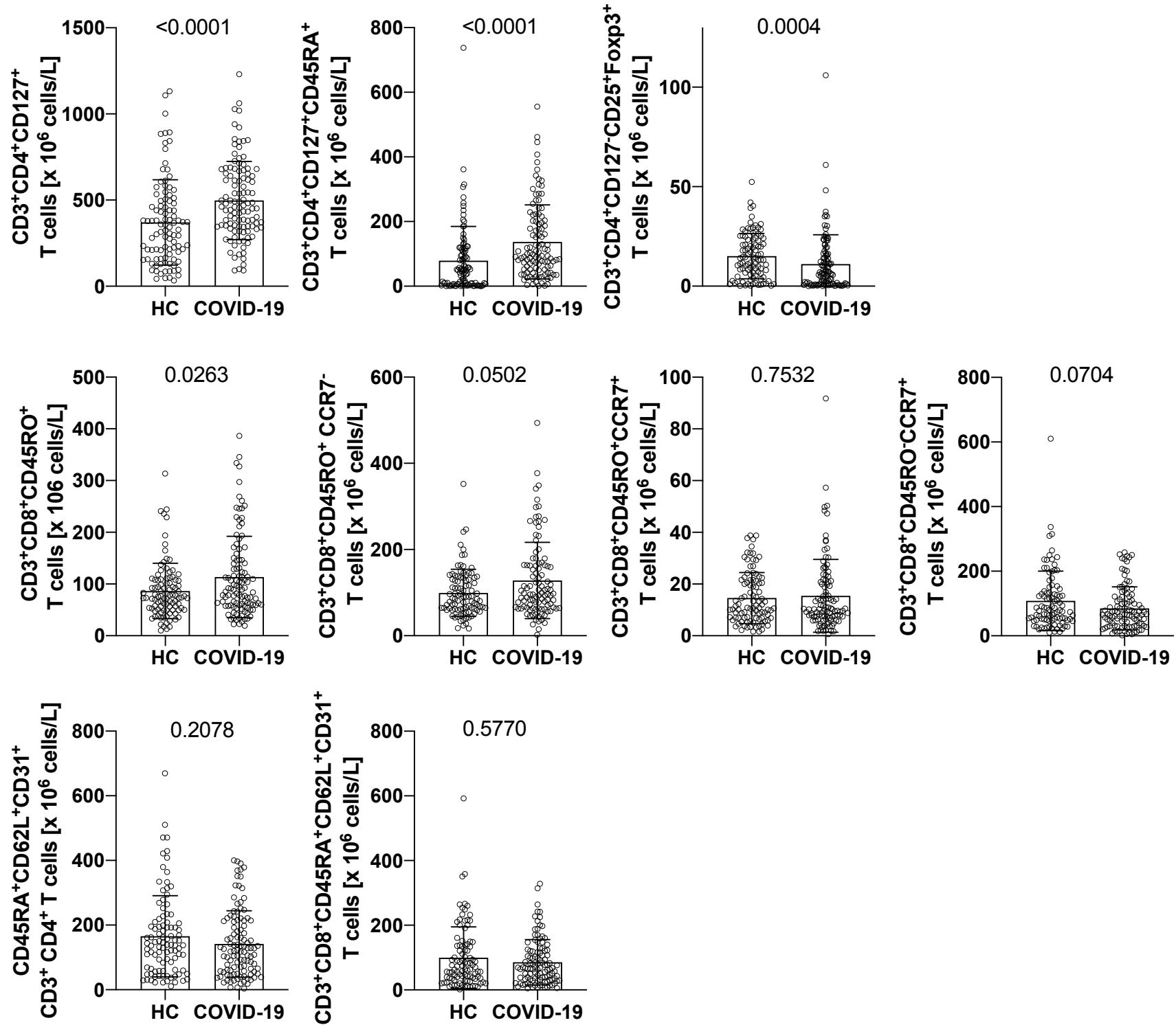

Supplement: Supplementary file 3 — Fig S3 [file ALL-76-751-s006.pdf]

FIGURE S4

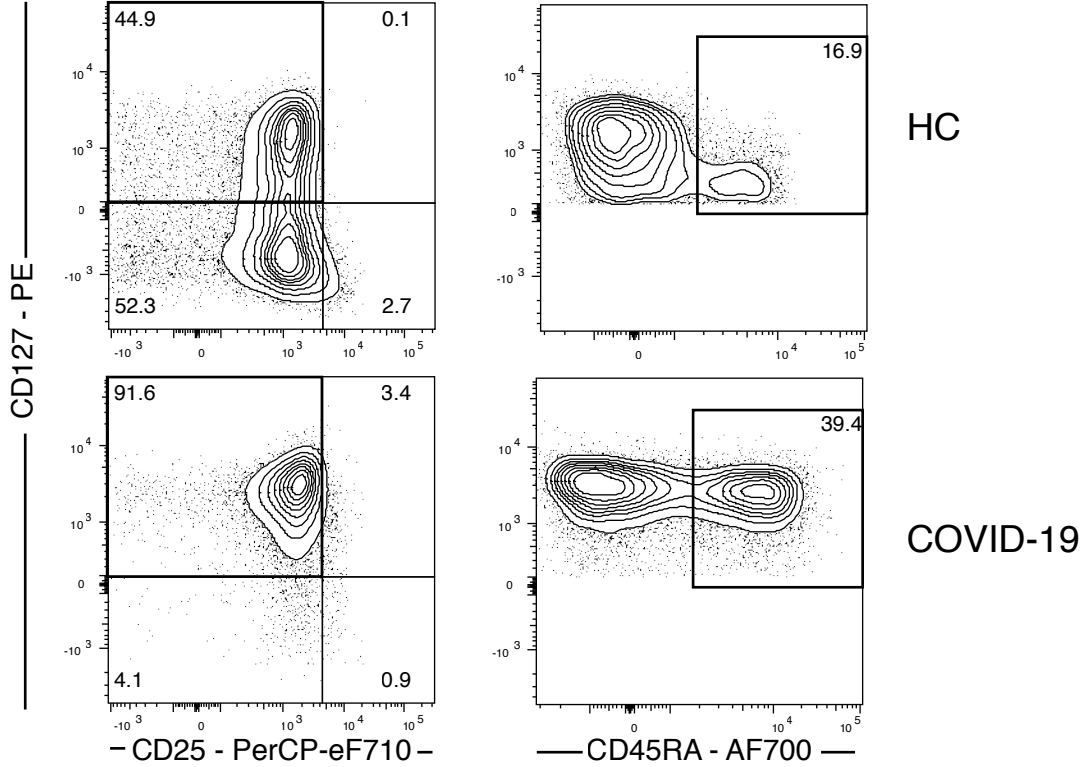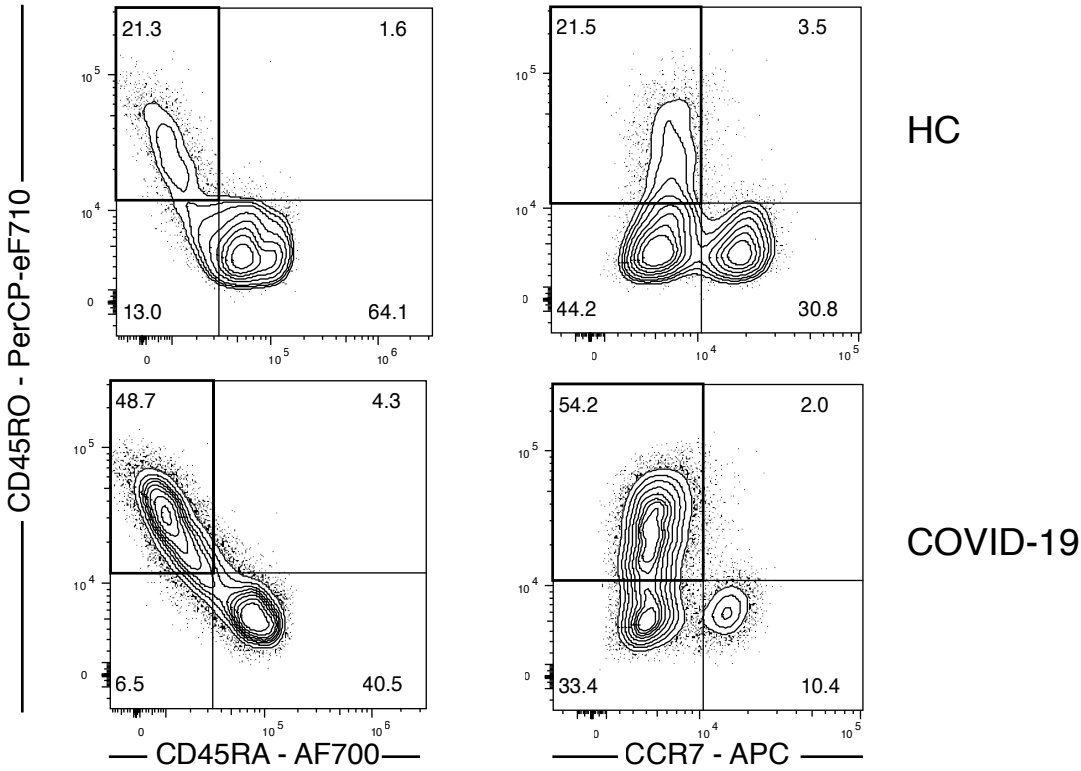

Supplement: Supplementary file 4 — Fig S4 [file ALL-76-751-s005.pdf]

FIGURE S5

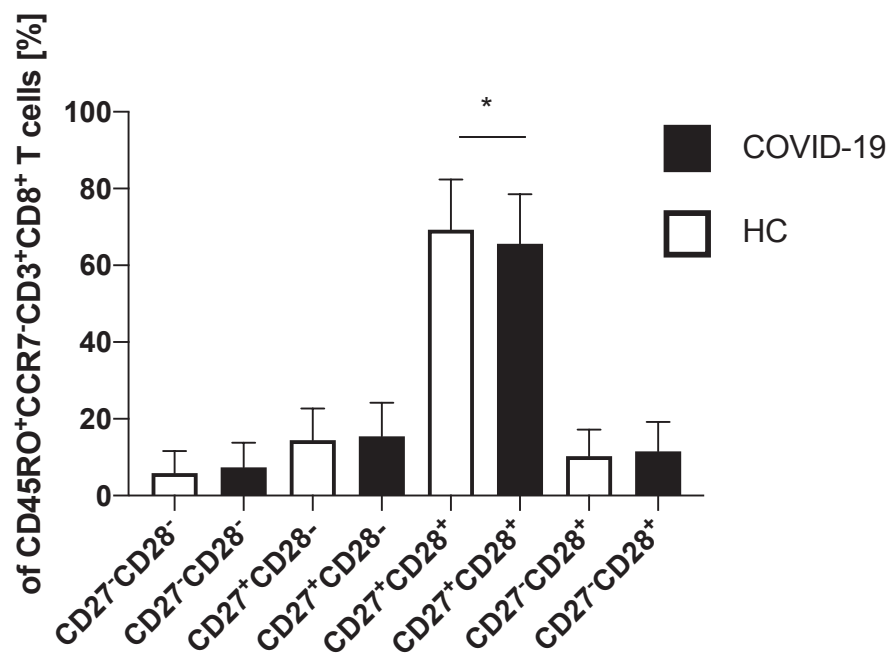

Supplement: Supplementary file 5 — Fig S5 [file ALL-76-751-s004.pdf]

FIGURE S6

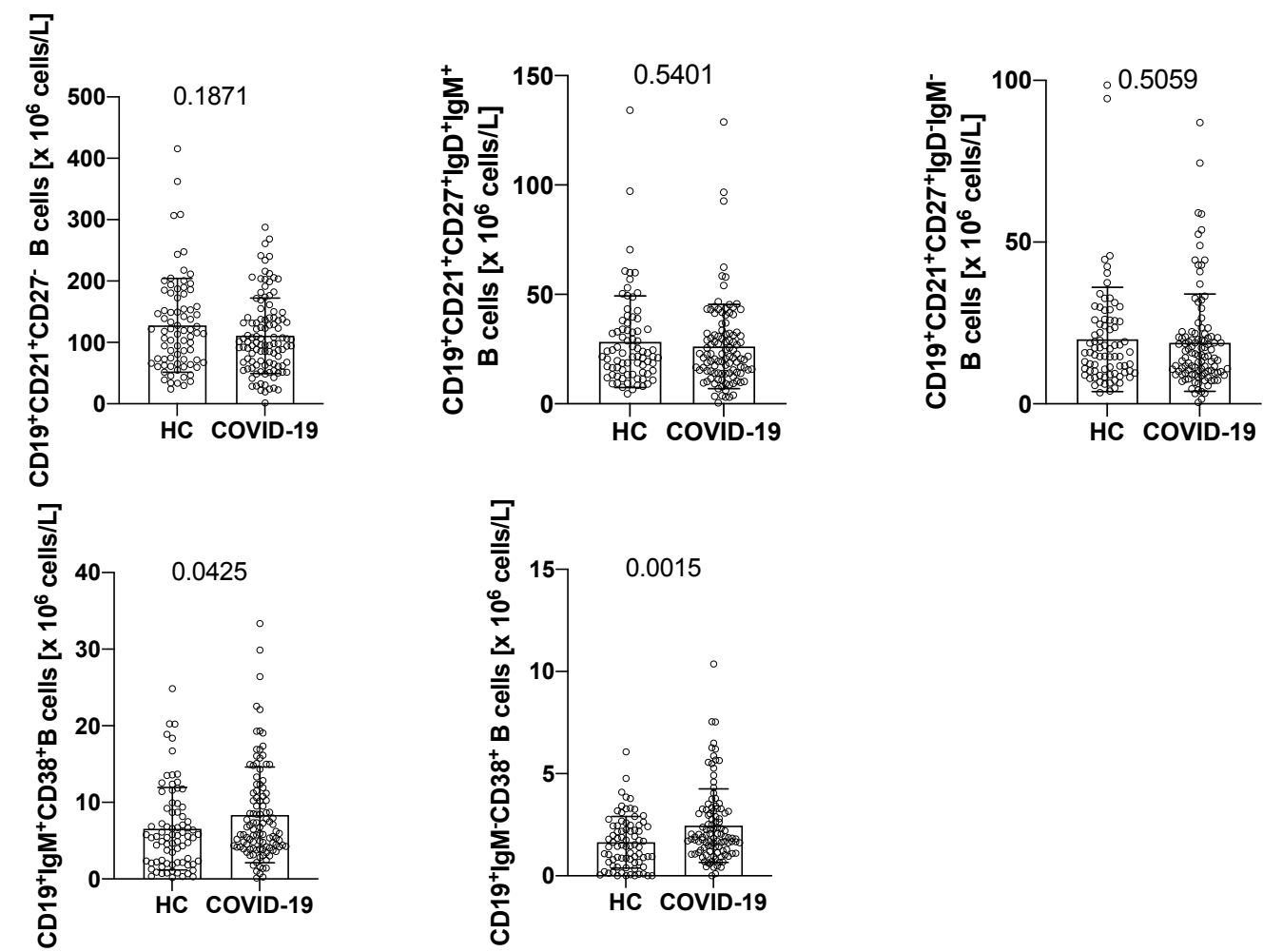

Supplement: Supplementary file 6 — Fig S6 [file ALL-76-751-s009.pdf]

FIGURE S7

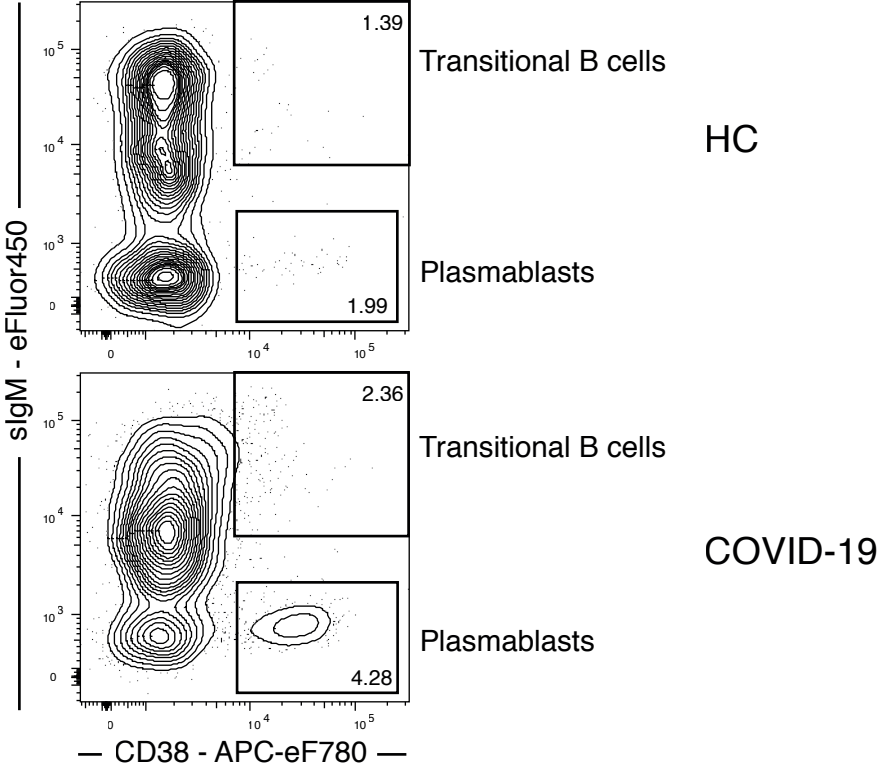

Supplement: Supplementary file 7 — Fig S7 [file ALL-76-751-s001.pdf]
