# Supplementary material for: Immunological imprint of COVID‐19 on human peripheral blood leukocyte populations
Source: Allergy. 2020 Nov 22;76(3):751–65. doi: 10.1111/all.14647 (PMC7984452; doi:10.1111/all.14647)
Supplement: Supplementary file 8 — Fig S8 [file ALL-76-751-s002.pdf]

FIGURE S8

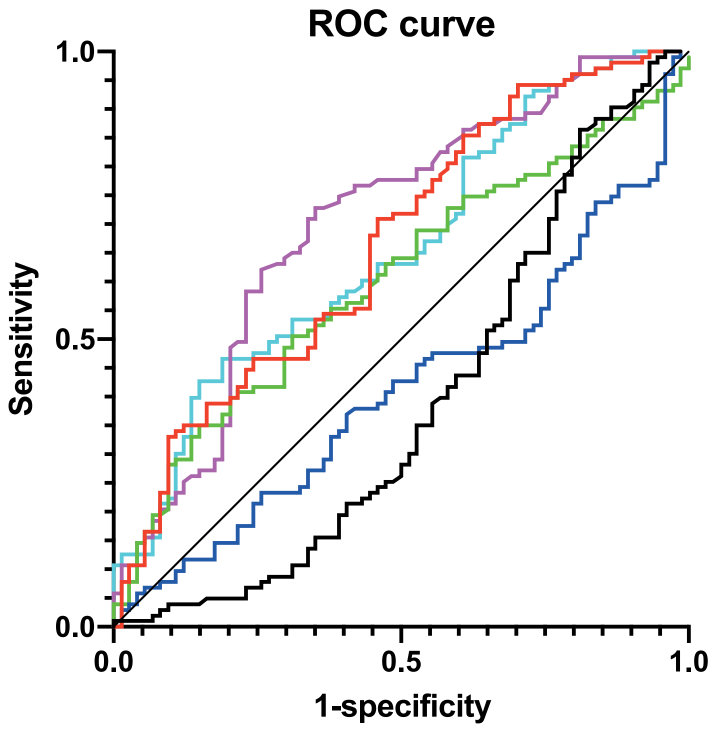

- %CD3^{+}CD8^{+}CD27^{+}CD28^{-}CCR7^{-}CD45RA$^{-/+}$ AE3 cells of CD3^{+}CD8^{+} T cells
- %CD3^{+}CD4^{+}CD127^{+}CD45RA^{+} of CD3^{+}CD4^{+} T cells
- %CD3^{+}CD4^{+}CD127^{-}CD25^{+}Foxp3^{+}CD45RA^{+} of CD3^{+}CD4^{+}CD127^{-}CD25^{+}T cells
- %CD3^{+}CD8^{+}CD45RO^{+}CCR7^{-} of CD3^{+}CD8^{+} T cells
- %CD19^{+}IgM^{-}CD38^{+} plasmablasts of CD19^{+} B cells
- %CD19^{+}IgM^{+}CD38^{+} transitional B cells of CD19^{+} B cells
