# Supplementary material for: Immunological imprint of COVID‐19 on human peripheral blood leukocyte populations
Source: Allergy. 2020 Nov 22;76(3):751–65. doi: 10.1111/all.14647 (PMC7984452; doi:10.1111/all.14647)
Supplement: Supplementary file 9 — Supplementary Material [file ALL-76-751-s003.docx]

**Appendix S1**

**Online Repository**

Bernhard Kratzer^1^, Doris Trapin^1^, Paul Ettel^1^, Ulrike Körmöczi^1^, Arno Rottal^1^, Friedrich Tuppy^1^, Melanie Feichter^1^, Pia Gattinger^2^, Kristina Borochova^2^, Yulia Dorofeeva^2^, Inna Tulaeva^2, 3^, Milena Weber^2^, Katharina Grabmeier-Pfistershammer^1^, Peter Tauber^1^, Marika Gerdov^4^, Bernhard Mühl^5^, Thomas Perkmann^4^, Ingrid Fae^6^, Sabine Wenda^6^, Harald Führer^7^, Rainer Henning^8^, Rudolf Valenta^2, 3, 9, 10, *^ and Winfried F. Pickl^1, *^

^1^Institute of Immunology, Center for Pathophysiology, Infectiology and Immunology, Medical University of Vienna, Vienna, Austria.

^2^Institute of Pathophysiology and Allergy Research, Center for Pathophysiology, Infectiology and Immunology, Medical University of Vienna, Vienna, Austria.

^3^Laboratory for Immunopathology, Department of Clinical Immunology and Allergology, I. M. Sechenov First Moscow State Medical University (Sechenov University), Moscow, Russia.

^4^Department of Laboratory Medicine, Medical University of Vienna, Vienna,

Austria.

^5^Labors.at, Vienna, Austria.

^6^ Department for Blood Group Serology and Transfusion Medicine, Medical University of Vienna, Vienna, Austria.

^7^Statistical Consultants, Vienna, Austria.

^8^Viravaxx, Vienna, Austria.

^9^NRC Institute of Immunology FMBA of Russia, Moscow, Russia.

^10^Karl Landsteiner University of Health Sciences, Krems, Austria.

***) Corresponding authors:**

Winfried F. Pickl, MD

Institute of Immunology, Center for Pathophysiology, Infectiology and Immunology, Medical University of Vienna, Lazarettgasse 19, 1090 Vienna, Austria.

Phone: (+431) 40160 33245.

Fax: (+431) 40160 933245.

Email: [winfried.pickl@meduniwien.ac.at](mailto:winfried.pickl@meduniwien.ac.at).

ORCID ID: orcid.org/0000-0003-0430-4952

&

Rudolf Valenta

Department of Pathophysiology and Allergy Research

Medical University of Vienna

Waehringer Guertel 18-20

A-1090 Vienna, Austria

Tel: (+431) 40400 51080

Fax: (+431) 40400 51300

E-mail: [rudolf.valenta@meduniwien.ac.at](mailto:rudolf.valenta@meduniwien.ac.at)

ORCID ID: 0000-0001-5944-3365

**SUPPLEMENTAL MATERIALS AND METHODS**

**Immunophenotyping by multiparametric flow cytometry**

Briefly, EDTA-anticoagulated blood was washed three times with PBS and 50 to 100 µl were incubated with optimal concentrations of the antibodies listed in **Table S1**.

Samples were incubated with the respective antibody mix for 15 minutes, followed by the addition of 100 µl Nordic lyse (Nordic MUbio, Susteren, The Netherlands) and incubated for 10 minutes. Subsequently, 4.5 ml of double distilled H_2_0 was added to each sample, incubated for another 5 minutes and centrifuged at 500 g for 5 minutes. Then, samples were acquired on a Navios Ex flow cytometer (Beckman Coulter, Brea, CA) equipped with three laser lines and analyzed with the Kaluza software package (Beckman Coulter).

Intracellular stainings were performed as described previously ^1^. Briefly, 100 µl of washed EDTA-anticoagulated blood was incubated with 2 ml of BD Pharm Lyse (Becton Dickinson, Franklin Lakes, NJ, USA) for 10 minutes and washed with PBS+0.5% BSA and 0.05% NaN_3_. Afterwards, optimal concentrations of the antibodies listed in **Table S1** for surface staining were incubated for 15 minutes and washed with PBS+0.5% BSA and 0.05% NaN_3_. Subsequently 100 µl of 1:4 diluted Fixation concentrate with Perm diluent (Thermo Fischer Scientific, Waltham, MA, USA) was added to the samples and incubated for 30 minutes, subsequently the samples were washed with 2 ml of permeabilization buffer. For intracellular staining, optimal concentrations of anti-Foxp-3 and anti-Helios mAbs were added for 30 minutes and samples were washed once with 2 ml of permeabilization buffer and once with 4.5 ml of permeabilization buffer. After the staining procedure, samples were acquired on a Navios Ex flow cytometer (Beckman Coulter, Brea, CA) equipped with three laser lines and analyzed with the Kaluza software package (Beckman Coulter).

**Real-time PCR assessment of TRECs and KRECs**

DNA was isolated from peripheral blood leukocytes using a Maxwell 16 DNA extraction device (Promega Corporation, Madison, WI) according to the manufacturer’s instructions. The quantity of TRECs and KRECs was assessed by real-time PCR (in-house validated using control collectives of different age (newborns, their mothers, penta- and heptagenarians) performed on a QuantStudio 6 Real-Time PCR System (Applied Biosystems, Waltham, MA). In brief, 1.25 µl of a DNA solution (concentration ranges between 25 and 70 ng per µl) were incubated in a mastermix (TaqMan Universal PCR Master Mix, Thermo Fisher Scientific, Waltham, MA), together with oligonucleotide primers and probes in a final volume of 25 µl. Probes were labelled at 5-’ and 3’-ends with the fluorescent dyes 6FAM or TAMRA. Reactions were setup in triplicates. Primer-probe combinations ^2^ were used to detect T cell receptor excision circles (forward primer 5’-CAC ATC CCT TTC AAC CAT GCT-3’, reverse primer 5’-TGC AGG TGC CTA TGC ATC A-3’, and probe 5’-ACA CCT CTG GTT TTT GTA AAG GTG CCC ACT-3’), kappa-deleting recombination excision circles (forward primer 5’-TCC CTT AGT GGC ATT ATT TGT ATC ACT-3’, reverse primer 5’-AGG AGC CAG CTC TTA CCCTAG AGT-3’, and probe 5’- TCT GCA CGG GCA GCA GGT TGG-3’) or the reference TR alpha constant gene (TRAC) (forward primer 5’-TGG CCT AAC CCT GAT CCT CTT-3’, reverse primer 5’-GGA TTT AGA GTC TCT CAG CTG GTA CAC-3’, and probe 5’-TCC CAC AGA TAT CCA GAA CCC TGA CCC-3’). PCR started with an initial incubation at 95 °C for eight minutes followed by 60 cycles of denaturation at 95 °C for 15 seconds annealing at 55 °C for 30 seconds and extension at 72 °C for 30 seconds. The threshold cycles (Ct-values) as reported by the machine were edited manually if necessary. The median Ct-value of each triplicate was used as relative measure of the concentration of the respective amplification product. The difference between the Ct-values of the TRAC gene and the Ct-values of the excision circle genes was used to calculate the number of excision circle containing cells per 1x10^5^ CD3^+^ or CD19^+^ lymphocytes, respectively, using the formula: 1 x 10^5^ x 2^Ct-reference+1−Ct-target^ x subpop^−1^, where Ct-reference denotes the threshold cycle of the reference (TRACs) amplification, Ct-target denotes the threshold cycle of either TRECs or KRECS amplification, respectively; subpop indicates the fraction of either CD3^+^ or CD19^+^ lymphocytes in comparison to all leukocytes. For the TRAC gene a correction of an additional cycle was applied to accommodate for the two copies present in either CD3^+^ or CD19^+^ lymphocytes. Data obtained with TRECs and KRECs analyses significantly correlated with numbers of RTE (positively, p =4.2 x 10^-15^) or memory T cells (negatively, p=2.4 x 10^-6^) and naive (positively, p=4.3 x 10^-7^) or memory B cells (negatively, p=5.4 x 10^-7^), respectively.

**Determination of SARS-CoV-2-specific serum antibodies**

SARS-CoV-2 antibody titers were analyzed as previously described ^9^ with the following modifications. IgG responses to SARS-CoV-2 S protein and the receptor binding domain (RBD) were determined by ELISA. SARS-CoV-2 S protein (Genscript, Piscataway, NJ, USA) and RBD (GenScript) were coated at 2 µg/ml in bicarbonate buffer onto NUNC Maxisorb 96 well plates (Thermofisher, Thermo-Fisher Scientific, Waltham, MA, USA) at room temperature for 5 hours. Plates were washed 3 times with washing buffer (PBS, 0.05% Tween 20) and subsequently blocked with blocking buffer (PBS, 0.05% Tween 20, 2% BSA) over night. Serum samples 1:40 diluted were applied and incubated at 4°C overnight. To determine human IgG reactivity, plates were washed 3 times and incubated with 1: 1000 diluted HRP-conjugated anti-human IgG (BD, San Jose, CA, USA) for 1 hour. After washing 3 times, the plates were developed with ABTS (Sigma-Aldrich, St. Louis, MO, USA) for colorimetric detection of bound antibodies and optical density was measured at 405/492 nm with an Infinite F50 ELISA reader (Tecan, Männedorf, Switzerland).

In addition, total antibodies against the SARS-CoV-2 nucleocapsid (N) antigen were measured with the Elecsys® Anti-SARS-CoV-2 assay (Roche Diagnostics, Rotkreuz, Switzerland) on a Cobas e 801 modular analyzer (Roche Diagnostics, Rotkreuz, Switzerland) as described previously ^34^.

**Statistical analyses**

We assessed variables potentially associated with COVID-19 disease. To test the H_0_ of no association with the outcome univariate analysis of differences between COVID-19 patients and healthy subjects was carried out with the Mann-Whitney-U-test due to outliers and skewness of some variables.

Multivariate logistic regression analyses were performed for the 46 blood cell and serum parameters, which indicated significant differences in the univariate analyses. For that purpose, forward stepwise logistic regression by Wald's statistic (Nagelkerkes R²=0,572, Omnibus-Test p<0.001) was performed and additional parameters were included as long as p<0.05. Principal component analyses (PCA) were performed using clinical parameters and by applying Bartlett's test of sphericity.

For data management MS Excel was used. Data were calculated using the SPSS Version 17.0 (IBM, New York, NY) and the GraphPad 6.0 software (GraphPad Software Inc., La Jolla, CA), respectively.

**SUPPLEMENTAL REFERENCES**

1. Kohler C, Smole U, Kratzer B, Trapin D, Schmetterer KG, Pickl WF. Allergen alters IL-2/alphaIL-2-based Treg expansion but not tolerance induction in an allergen-specific mouse model. *Allergy.* 2020;75(7):1618-1629.

2. Sottini A, Ghidini C, Zanotti C, et al. Simultaneous quantification of recent thymic T-cell and bone marrow B-cell emigrants in patients with primary immunodeficiency undergone to stem cell transplantation. *Clin Immunol.* 2010;136(2):217-227.

**SUPPLEMENTAL FIGURE AND TABLE LEGENDS**

**TABLE S1.** List of monoclonal antibodies used in this study.

**TABLE S2.** List of regular medications taken by COVID-19 convalescent patients and healthy control subjects

**TABLE S3.** Significant differences in leukocyte populations between COVID-19 convalescent patients and healthy control subjects.

**TABLE S4.** Significant differences in T lymphocyte subpopulations between COVID-19 convalescent patients and healthy control subjects.

**TABLE S5.** Significant differences in B lymphocyte subpopulations between COVID-19 convalescent patients and healthy control subjects.

**FIGURE S1.** Impact of primary SARS-CoV-2 infection on absolute counts of leukocyte subpopulations as determined 10 weeks after disease onset. Shown are relative values of the indicated leukocyte populations in peripheral blood of healthy control subjects (HC) and COVID-19 convalescent patients (COVID-19). Bars show mean values, whiskers the standard deviation, and open circles the values of single individuals. Data show pooled results of daily stainings (whole blood, 6-9 individuals per day) of n=98 for HC, and n=109 for COVID-19 patients, except for panels determining HLA-DR expression where n=97 for HC. P-values were determined by Mann-Whitney U-test and are indicated.

**FIGURE S2.** Primary SARS-CoV-2 infection leads to significant and sustained activation of CD3^+^CD8^+^ T Lymphocytes**.** Shown are representative two-parameter contour blots of a healthy control subject and a COVID-19 convalescent patient stained for CD3, CD4 and CD8 (shown on the x-axis, respectively) and HLA-DR (y-axis). Markers were set according to negative lymphoid cell populations. Numbers indicate percentage of cells within the respective quadrant.

**FIGURE S3.** Impact of primary SARS-CoV-2 infection on absolute amounts of T cell subpopulations levels as determined 10 weeks after disease onset. Shown are the relative values of selected T cell subpopulations in PB of healthy control subjects (HC) and COVID-19 convalescent patients (COVID-19). Bars show mean values, whiskers the standard deviation, and open circles the values of single individuals. Data show pooled results of daily stainings (whole blood, 5-9 individuals per day) of n=98 for HC, and n=109 for COVID-19 patients, except for panels determining CD25/Foxp3 n=97 for HC; and CD45RO/CCR7 n=95 for HC and n=105 for COVID-19. P-values were determined by Mann-Whitney U-test and are indicated.

**FIGURE S4.** Primary SARS-CoV-2 infection significantly increases the number of CD4^+^CD127^+^ and of CD3^+^CD8^+^CD45RA^-^CD45RO^+^CCR7^+^ T Lymphocytes. **A)** Shown are representative two-parameter contour blots of a healthy control subject and a COVID-19 convalescent patient stained for CD3, CD4 (gate), CD25 or CD45RA (x-axis) and CD127 (y-axis). Markers were set according to negative lymphoid cell populations. **B)** Shown are two-parameter contour blots of a representative healthy donor and a representative COVID-19 convalescent individual stained for CD3, CD8 (gate), CD45RA or CCR7 (x-axis) and CD45RO (y-axis). Markers were set according to negative lymphoid cell populations. Numbers indicate percentage of cells within the respective quadrant.

**FIGURE S5.** Frequency of CD27 and CD28 expression on CD3+CD8+CD45RO+CCR7+ memory T cells. Shown are the mean frequencies of CD27 and/or CD28 expression on cytotoxic memory T cells of COVID-19 convalescent patients (black bars) as compared to healthy control subjects (white bars). Whiskers show the standard deviation. *, P < 0.05 as determined by ANOVA

**FIGURE S6.** Impact of primary SARS-CoV-2 infection on absolut amounts of B cell subpopulations as determined 10 weeks after disease onset. Shown are the relative values of the indicated B cell sub populations in PB of healthy control subjects (HC) and COVID-19 convalescent patients (COVID-19). Bars show mean values, whiskers the standard deviation, and open circles the values of single individuals. Data show pooled results of daily stainings (whole blood, 5-9 individuals per day) of n=78 for HC, and n=108 for COVID-19 patients. P-values were determined by Mann-Whitney U-test and are indicated.

**FIGURE S7.** Primary SARS-CoV-2 infection leads to significant and sustained increases of both transitional B cells and plasmablasts. Shown are representative two-parameter contour blots of a healthy control subject and a COVID-19 convalescent patients stained for CD19 (gate) and CD38 (x-axis) and surface IgM (y-axis). Markers were set according to negative lymphoid cell populations. Numbers indicate percentage of cells within the respective quadrant.

**FIGURE S8.** ROC statistics of blood parameters. This subset model is the result of forward stepwise logistic regression by Wald's statistic (Nagelkerkes R²=0,572, Omnibus-Test p<0.000). Additional parameters were included as long as p < 0.05.

**TABLE S1. List of monoclonal antibodies used for flow cytometry for analyses of cell surface and intracellular antigens.**

| Specificity | Clone  Name | Species | Fluorophore | Source |
| --- | --- | --- | --- | --- |
| CD8 | RPA-T8 | Mouse IgG1 | FITC | Thermo Fisher Scientific, Waltham, MA, USA |
| sIgK | TB28-2 | Mouse IgG1 | FITC | Thermo Fisher Scientific |
| TCR a/b | WT31 | Mouse IgG1 | PE | Thermo Fisher Scientific |
| CD19 | HIB19 | Mouse IgG1 | PE-eFluor610 | Thermo Fisher Scientific |
| CD4 | RPA-T4 | Mouse IgG1 | PE-eFluor610 | Thermo Fisher Scientific |
| CD33 | WM-53 | Mouse IgG1 | PerCP-Cy5.5 | Thermo Fisher Scientific |
| CD3 | UCHT1 | Mouse IgG1 | PE-Cy7 | Thermo Fisher Scientific |
| CD5 | L17F12 | Mouse IgG2a | APC | Thermo Fisher Scientific |
| CD20 | 2H7 | Mouse IgG2b | AF700 | Thermo Fisher Scientific |
| CD38 | HIT2 | Mouse IgG1 | APC-eFluor780 | Thermo Fisher Scientific |
| HLA-DR | LN3 | Mouse IgG2b | eFluor450 | Thermo Fisher Scientific |
| CD45 | HI30 | Mouse IgG1 | eFluor506 | Thermo Fisher Scientific |
| sIgD | IA6-2 | Mouse IgG2a | FITC | Thermo Fisher Scientific |
| CD21 | HB5 | Mouse IgG2a | PE | Thermo Fisher Scientific |
| CD10 | SN5c | Mouse IgG1 | PerCP-eFluor710 | Thermo Fisher Scientific |
| CD27 | O323 | Mouse IgG1 | PE-Cy7 | Thermo Fisher Scientific |
| CD24 | eBioSN3 (SN3 A5-2H10) | Mouse IgG1 | APC | Thermo Fisher Scientific |
| sIgM | SA-DA4 | Mouse IgG1 | eFluor450 | Thermo Fisher Scientific |
| CD27 | O323 | Mouse IgG1 | FITC | Biolegend, San Diego, CA, USA |
| CD62L | SK11 | Mouse IgG2a | PE | Becton Dickinson, Franklin Lakes, NJ, USA |
| CD45RO | UCHL1 | Mouse IgG2a | PerCP-eFluor710 | Thermo Fisher Scientific |
| CD197 (CCR7) | 3D12 | Rat IgG2a | APC | Thermo Fisher Scientific |
| CD45RA | HI100 | Mouse IgG2b | AF700 | Biolegend |
| CD28 | CD28.2 | Mouse IgG1 | APC-eFluor780 | Thermo Fisher Scientific |
| CD8 | RPA-T8 | Mouse IgG1 | eFluor450 | Thermo Fisher Scientific |
| CD31 | WM-59 | Mouse IgG1 | BV510 | Becton Dickinson |
| Foxp3 | PCH101 | Rat IgG2a | FITC | Thermo Fisher Scientific |
| CD127 | eBioRDR5 | Mouse IgG1 | PE | Thermo Fisher Scientific |
| CD25 | CD25-4E3 | Mouse IgG2b | PerCP-eFluor710 | Thermo Fisher Scientific, |
| Helios | 22F6 | Hamster IgG | APC | Thermo Fisher Scientific |
| CD73 | AD2 | Mouse IgG1 | APC-eFluor780 | Thermo Fisher Scientific |
| CD39 | eBioA1 | Mouse IgG1 | Super Bright 436 | Thermo Fisher Scientific |

Table shows the specificity, clone names, species and supplier (source) of the respective monoclonal antibodies and their conjugation with the respective fluorophores used in this study.

**TABLE S2. List of drugs reported by the patients**

| Drug | COVID-19 convalescent patients (n=108) | Healthy control  subjects (n=98) |
| --- | --- | --- |
| abacavir, dolutegravir, lamivudine | 1 | 0 |
| acetylsalicylic acid | 9 | 3 |
| allopurinol | 0 | 1 |
| amiodarone | 1 | 0 |
| amlodipine | 5 | 3 |
| apixaban | 1 | 0 |
| atorvastatin | 3 | 2 |
| atovaquone | 1 | 0 |
| beclomethasone dipropionate | 0 | 1 |
| bisoprolol | 5 | 1 |
| budesonide | 2 | 3 |
| Ca^2+^; vitamin D3 | 1 | 0 |
| cabergoline | 0 | 1 |
| candesartan | 5 | 5 |
| cyclosporine | 1 | 0 |
| dabigatran | 0 | 1 |
| daflon | 1 | 0 |
| dapagliflozin | 1 | 0 |
| desloratadine | 1 | 1 |
| diosmin | 0 | 1 |
| doxazosin | 1 | 0 |
| edoxaban | 1 | 0 |
| empagliflozin | 1 | 0 |
| enalapril | 1 | 3 |
| erenumab | 1 | 0 |
| escitalopram | 0 | 1 |
| esomeprazole | 1 | 1 |
| estradiol | 0 | 1 |
| estriol | 0 | 1 |
| ethinylestradiol | 1 | 2 |
| ezetimibe | 1 | 0 |
| famotidine | 1 | 0 |
| fenofibrate | 1 | 0 |
| fexofenadine | 1 | 0 |
| finasteride | 1 | 0 |
| fluoxetine | 0 | 1 |
| fluticasone | 0 | 1 |
| fluvoxamine | 0 | 1 |
| formoterol | 3 | 4 |
| guanfacine | 1 | 0 |
| hydrochlorothiazide | 2 | 2 |
| ibandronic acid | 0 | 1 |
| imidapril | 0 | 1 |
| insulin | 1 | 0 |
| irbesartan | 1 | 0 |
| Iron(II)fumarate | 2 | 0 |
| lamotrigine | 1 | 0 |
| hydrochloride lercanidipine | 1 | 1 |
| levocetirizine, cetirizine-AD6C | 0 | 3 |
| levonorgestrel | 0 | 1 |
| levothyroxine | 12 | 13 |
| linagliptin | 1 | 0 |
| lipase-, amylase-, protease-mix | 0 | 1 |
| lisinopril | 3 | 1 |
| magnesium | 1 | 0 |
| mefenamic acid | 0 | 1 |
| metamizole | 2 | 1 |
| metformin | 2 | 1 |
| methylphenidate | 0 | 1 |
| methylprednisolone | 2 | 1 |
| metoprolol | 2 | 0 |
| montelukast | 3 | 1 |
| naproxen | 1 | 0 |
| nebivolol | 2 | 1 |
| olodaterol | 0 | 1 |
| pantoprazol | 1 | 2 |
| paroxetine | 1 | 0 |
| phenoxymethylpenicillin | 1 | 0 |
| phenprocoumon | 0 | 1 |
| pregabalin | 1 | 1 |
| ramipril | 2 | 0 |
| rilmenidine | 1 | 0 |
| rosuvastatin | 2 | 2 |
| salmeterol | 0 | 1 |
| sertraline | 1 | 2 |
| simvastatin | 2 | 1 |
| tamsulosin | 4 | 0 |
| terazosin | 1 | 0 |
| testosterone | 0 | 1 |
| tiotropium bromide | 0 | 1 |
| tramadol | 0 | 1 |
| trazodone | 2 | 0 |
| triazolam | 1 | 0 |
| trimetazidine | 1 | 0 |
| valsartan | 3 | 1 |
| venlafaxine | 1 | 1 |
| verapamil | 1 | 0 |
| vitamin B9 folic acid | 1 | 0 |
| vitamin D3 | 3 | 3 |
| zolpidem | 1 | 0 |

Shown are the numbers of patients and control subjects taking the indicated drugs

**TABLE S3. Significant differences in leukocyte populations between COVID-19 convalescent patients and healthy control subjects.**

|  | **COVID-19 convalescent patients (n=109) (absolute counts)** | **Healthy control subjects (n=98) (absolute counts)** | **P COVID-19 vs HC** | **COVID-19 convalescent patients (n=109) (relative values)** | **Healthy control subjects (n=98) (relative values)** | **P COVID-19 vs HC** |
| --- | --- | --- | --- | --- | --- | --- |
| Leukocytes | 6,384±1.677^(*^ | 7,006±1,961 | 0.0156^(§^ |  |  |  |
| Granulocytes | 4,111±1,477 | 4,693±1,588 | 0.0033 | 63.31±9.39 | 66.40±8.77 | 0.0239 |
| Monocytes | 478±159 | 468±207 | 0.5190 | 7.63±2.17 | 6.70±2.14 | 0.0177 |
| Lymphocytes | 1,809±571 | 1,857±622 | 0.5449 | 29.10±8.22 | 27.08±7.71 | 0.0590 |
| CD3+ T cells | 1,324±461 | 1,338±472 | 0.8162 | 72.47±7.93 | 71.84±7.11 | 0.4529 |
| CD3+TCR ab+ T cells | 1248±440 | 1258±449 | 0.8781 | 68.31±7.85 | 67.50±7.15 | 0.3406 |
| CD3+ TCR gd+ T cells | 69±70 | 69±65 | 0.7369 | 3.79±3.58 | 3.65±3.24 | 0.8487 |
| CD3+CD4+ T cells | 807±291 | 846±332 | 0.5411 | 44.65±8.55 | 45.46±7.78 | 0.6100 |
| CD3+CD8+ T cells | 428±242 | 402±195 | 0.7573 | 22.98±8.65 | 21.61±7.11 | 0.3574 |
| CD4/CD8-Ratio | 2.32±1.18 | 2.46±1.33 | 0.4349 |  |  |  |
| CD3+CD4+CD8+ DP T cells | 19±32 | 19±34 | 0.2969 | 0.98±1.37 | 0.98±1.46 | 0.4424 |
| CD3+TCR ab+ CD4-CD8- T cells | 22±19 | 23±22 | 0.6089 | 1.77±1.46 | 1.92±1.58 | 0.5303 |
| CD3+HLA-DR+ T cells | 107±85 | 81±54 | 0.0248 | 5.78±3.84 | 4.34±2.50 | 0.0054 |
| CD3+CD4+HLA-DR+ T cells | 33±18 | 31±19 | 0.3630 | 1.88±0.93 | 1.70±0.87 | 0.1921 |
| CD3+CD8+HLA-DR+ T cells | 61±65 | 38±34 | 0.0079 | 3.21±2.91 | 2.05±1.61 | 0.0014 |
| CD3+HLA-DR+CD38+ T cells | 4.6±3.6 | 3.5±2.7 | 0.0275 | 0.27±0.24 | 0.19±0.14 | 0.0051 |
| CD3+CD4+HLA-DR+CD38+ T cells | 2.1±1.6 | 1.6±1.2 | 0.0605 | 0.12±0.12 | 0.09±0.06 | 0.0210 |
| CD3+CD8+HLA-DR+CD38+ T cells | 1.5±1.7 | 1.0±1.2 | 0.0473 | 0.09±1.06 | 0.05±0.07 | 0.0178 |
| CD19+ B cells | 175±83 | 187±99 | 0.6873 | 9.69±3.32 | 10.03±3.62 | 0.6787 |
| CD3-CD56+ NK cells | 251±124 | 261±144 | 0.9870 | 14.46±7.52 | 14.17±6.21 | 0.9842 |
| CD3+CD56+ NKT cells | 8±11 | 8±14 | 0.4376 | 0.39±0.46 | 0.43±0.59 | 1.0000 |

^*)^ x 10^6^ cells/L as mean ± standard deviation

^§)^ significant P-values are shown in red font**TABLE S4. Significant differences in T lymphocyte subpopulations between COVID-19 convalescent patients and healthy control subjects.**

|  | **COVID-19 convalescent patients (n=109) (absolute counts)** | **Healthy control subjects (n=98) (absolute counts)** | **P COVID-19 vs HC** | **COVID-19 convalescent patients (n=109) (relative values)** | **Healthy control subjects (n=98) (relative values)** | **P COVID-19 vs HC** |
| --- | --- | --- | --- | --- | --- | --- |
| CD45RA+CD62L+CD31+ RTE in CD3+ T cells | 227±157 | 256±199 | 0.3222 | 16.85±10.35 | 19.11±11.13 | 0.1351 |
| CD4+CD45RA+CD62L+CD31+ RTE in CD3+CD4+ T cells | 141±103 | 165±126 | 0.2078 | 17.01±10.85 | 18.40±10.27 | 0.2215 |
| CD8+CD45RA+CD62L+CD31+ RTE in CD3+CD8+ T cells | 86±70 | 100±95 | 0.5770 | 20.81±13.78 | 23.73±16.64 | 0.3592 |
| CD127+ in CD3+CD4+ T cells | 498±226 | 370±248 | <0.0001 | 62.44±17.69 | 44.83±24.22 | <0.0001 |
| CD45RA+ naive T cells in CD3+ T cells | 479±271 | 539±279 | 0.0543 | 35.35±12.80 | 38.89±12.19 | 0.0350 |
| CD25+ in CD3+ T cells | 14±14 | 12±8 | 0.0872 | 1.12±0.92 | 0.90±0.65 | 0.0284 |
| CD4+CD45RA+ naive T cells in CD3+ T cells | 226±139 | 282±188 | 0.0309 | 17.00±8.34 | 20.21±9.32 | 0.0128 |
| CD45RA+CCR7+ in CD3+CD4+ T cells | 259±145 | 304±207 | 0.2283 | 31.00±12.70 | 33.83±12.82 | 0.1707 |
| CD45RO-CCR7+ in CD3+CD4+ T cells | 317±189 | 371±242 | 0.1577 | 37.77±14.57 | 41.03±13.18 | 0.1206 |
| CD45RA+CCR7- in CD3+CD4+ T cells | 43±42 | 59±72 | 0.2527 | 5.46±5.03 | 7.09±7.69 | 0.2462 |
| CD45RO-CCR7- in CD3+CD4+ T cells | 86±66 | 107±77 | 0.0150 | 11.35±10.79 | 12.66±7.11 | 0.0171 |
| CD4+CD27+CD28+CCR7+CD45RA+ in CD3+CD4+ naïve T cells | 220±140 | 261±187 | 0.2108 | 26.15±11.78 | 28.67±11.56 | 0.1372 |
| CD127+CD45RA+ in CD3+CD4+ T cells | 137±114 | 79±106 | <0.0001 | 24.47±13.68 | 15.66±13.79 | <0.0001 |
| CD127-CD25+ in CD3+CD4+ T cells | 39±27 | 38±22 | 0.6962 | 4.75±2.30 | 4.53±1.77 | 0.7230 |
| CD8+CD45RA+ T cells in CD3+ T cells | 225±173 | 225±136 | 0.4041 | 16.24±8.57 | 16.93±7.41 | 0.5365 |
| CD45RA+CCR7+ in CD3+CD8+ T cells | 83±67 | 101±89 | 0.1465 | 20.85±14.14 | 24.81±14.85 | 0.0633 |
| CD45RO-CCR7+ in CD3+CD8+ T cells | 85±66 | 108±92 | 0.0704 | 21.52±13.91 | 26.60±15.13 | 0.0145 |
| CD45RA+CCR7- in CD3+CD8+ T cells | 123±14 | 102±88 | 0.8420 | 25.35±14.74 | 24.70±14.95 | 0.7311 |
| CD45RO-CCR7- in CD3+CD8+ T cells | 195±184 | 175±136 | 0.8804 | 41.98±17.28 | 42.35±16.75 | 0.7973 |
| CD8+CD27+CD28+CCR7+CD45RA+ naïve cells in CD3+CD8+ T cells | 75±80 | 59±55 | 0.1952 | 16.93±11.44 | 14.71±10.81 | 0.1650 |
| CD45RO+ memory T cells in CD3+ T cells | 563±248 | 505±211 | 0.1178 | 43.53±13.00 | 39.26±12.68 | 0.0135 |
| CD4+CD45RO+ memory T cells in CD3+ T cells | 418±178 | 389±164 | 0.3109 | 32.56±10.15 | 30.23±9.63 | 0.0978 |
| CD45RO+CCR7- in CD3+CD4+ T cells | 251±129 | 221±88 | 0.1465 | 32.07±11.75 | 28.13±9.94 | 0.0075 |
| CD45RA-CCR7- in CD3+CD4+ T cells | 309±146 | 286±122 | 0.3431 | 39.47±13.17 | 35.84±12.05 | 0.0753 |
| CD4+CD27+CD28+CCR7+CD45RA- AE1 cells in CD3+CD4+ T cells | 175±94 | 180±111 | 0.8343 | 21.55±7.27 | 20.97±8.62 | 0.5200 |
| CD4+CD27+CD28+CCR7-CD45RA- AE2 cells in CD3+CD4+ T cells | 219±10 | 206±92 | 0.5368 | 27.75±9.56 | 25.79±9.01 | 0.1910 |
| CD8+CD45RO+ T cells in CD3+ T cells | 113±79 | 87±54 | 0.0263 | 8.58±4.67 | 6.82±3.88 | 0.0045 |
| CD45RO+CCR7- in CD3+CD8+ T cells | 128±89 | 99±55 | 0.0502 | 32.42±14.65 | 26.90±11.11 | 0.0052 |
| CD45RA-CCR7- in CD3+CD8+ T cells | 203±122 | 179±103 | 0.1695 | 49.80±15.14 | 46.23±15.05 | 0.1468 |
| CD8+CD27+CD28+CCR7+CD45RA- AE1 cells in CD3+CD8+ T cells | 15±20 | 10±10 | 0.0572 | 3.38±2.96 | 2.60±1.71 | 0.0974 |
| CD8+CD27+CD28+CCR7-CD45RA- AE2 cells in CD3+CD8+ T cells | 103±70 | 98±65 | 0.7532 | 24.36±10.17 | 25.34±10.77 | 0.5862 |
| CD45RO+CCR7+ in CD3+CD4+ T cells | 152±86 | 149±83 | 0.7109 | 18.81±7.69 | 18.18±8.47 | 0.2024 |
| CD45RA-CCR7+ in CD3+CD4+ T cells | 194±102 | 199±121 | 0.7944 | 24.07±8.23 | 23.23±9.38 | 0.4818 |
| CD4+CD27-CD28+CCR7-CD45RA-/+ AE3 cells in CD3+CD4+ T cells | 47±33 | 41±22 | 0.3406 | 6.05±3.40 | 5.12±2.53 | 0.0822 |
| CD4+CD27-CD28-CCR7-CD45RA-/+ AE4 cells in CD3+CD4+ T cells | 19±36 | 12±15 | 0.0912 | 2.47±3.91 | 1.57±2.01 | 0.0769 |
| CD45RO+CCR7+ in CD3+CD8+ T cells | 15±14 | 15±10 | 0.7532 | 4.08±3.06 | 4.14±3.21 | 0.9094 |
| CD45RA-CCR7+ in CD3+CD8+ T cells | 16±16 | 15±12 | 0.7440 | 4.00±3.02 | 4.26±3.83 | 0.7292 |
| CD8+CD27+CD28-CCR7-CD45RA-/+ AE3 cells in CD3+CD8+ T cells | 67±51 | 69±43 | 0.3686 | 15.68±7.31 | 18.15±8.99 | 0.0437 |
| CD8+CD27-CD28-CCR7-CD45RA-/+ AE4 cells in CD3+CD8+ T cells | 57±58 | 66±83 | 0.8554 | 13.83±10.97 | 15.17±12.70 | 0.6126 |
| CD8+CD27-CD28-CCR7-CD45RA+ TEMRA in CD3+CD8+ T cells | 28±29 | 33±47 | 0.6464 | 6.73±5.84 | 6.64±8.07 | 0.9201 |
| Foxp3+ in CD3+CD4+ CD127-CD25+T cells | 11±15 | 15±11 | 0.0004 | 27.73±25.70 | 42.10±27.26 | 0.0001 |
| Foxp3+CD39+ in CD3+CD4+ CD127-CD25+T cells | 8±13 | 10±9 | 0.0053 | 19.84±20.02 | 29.01±22.83 | 0.0012 |
| Foxp3+CD73+ in CD3+CD4+ CD127-CD25+T cells | 1.5±1.9 | 3.1±3.3 | <0.0001 | 3.45±3.84 | 7.69±6.88 | <0.0001 |
| Foxp3+Helios+ in CD3+CD4+ CD127-CD25+T cells | 8±13 | 10±8 | 0.0006 | 19.95±20.08 | 29.30±21.80 | 0.0011 |
| Foxp3+CD45RA+ in CD3+CD4+ CD127-CD25+T cells | 4±6 | 6±6 | 0.0023 | 11.18±11.07 | 16.17±13.85 | 0.0019 |

^*)^ x 10^6^ cells/L as mean ± standard deviation

^§)^ significant P-values are shown in red font

TEMRA T effector memory CD45RA^+^ T cells

AE antigen experienced T cells according to *Appay et al.* ^37^

**TABLE S5. Significant differences in B lymphocyte subpopulations between COVID-19 convalescent patients and healthy control subjects.**

|  | **COVID-19 convalescent patients (n=109) (absolute counts)^(*^** | **Healthy control subjects (n=98) (absolute counts)** | **P COVID-19 vs HC** | **COVID-19 convalescent patients (n=109) (relative values)** | **Healthy control subjects (n=98) (relative values)** | **P COVID-19 vs HC** |
| --- | --- | --- | --- | --- | --- | --- |
| CD20+ B cells^($^ | 170±83 | 180±98 | 0.8504 | 9.36±3.34 | 9.60±3.56 | 0.8522 |
| CD5+ B cells | 41±32 | 37±34 | 0.2374 | 2.25±1.61 | 2.02±1.68 | 0.1437 |
| Ig kappa+ B cells | 98±47 | 107±59 | 0.6122 | 5.42±1.91 | 5.68±2.26 | 0.6920 |
| Ig lambda+ B cells | 77±38 | 80±42 | 0.8893 | 4.24±1.52 | 4.28±1.62 | 0.9840 |
| CD21- B cells | 16±10 | 19±14 | 0.2397 | 9.87±4.41 | 10.23±5.95 | 0.7551 |
| CD21-CD10+ immature B cells | <0±0 | <0±0 | 0.2087 | 0.26±0.29 | 0.29±0.29 | 0.4330 |
| IgM+CD38+ transitional B cells | 8±6 | 7±5 | 0.0425^($^ | 5.00±3.07 | 3.57±2.30 | 0.0020 |
| CD21+CD27- naive B cells | 111±61 | 128±76 | 0.1871 | 62.71±12.80 | 62.95±13.26 | 0.8891 |
| CD21+CD27+ memory B cells | 47±31 | 51±33 | 0.4348 | 27.43±10.33 | 26.82±10.45 | 0.7228 |
| CD21+CD27+IgD+IgM+ non-switched memory B cells | 26±19 | 28±21 | 0.5401 | 15.13±7.16 | 14.73±6.85 | 0.7053 |
| CD21+CD27+IgD-IgM- switched memory B cells | 19±15 | 20±16 | 0.5059 | 11.07±5.71 | 10.52±5.80 | 0.3937 |
| CD21lowCD38low activated B cells | 7±6 | 9±10 | 0.1719 | 4.34±2.45 | 4.90±3.91 | 0.8028 |
| IgM-CD38+ plasmablasts | 2.5±1.8 | 1.6±1.3 | 0.0015 | 1.56±0.97 | 1.04±0.95 | <0.0001 |

^$)^ gated on CD19+ cells

^*)^ x 10^6^ cells/L as mean ± standard deviation

^§)^ significant P-values are shown in red font
